# Prognostic role of Gli1 expression in solid malignancies: a meta-analysis

**DOI:** 10.1038/srep22184

**Published:** 2016-02-22

**Authors:** Ji Cheng, Jinbo Gao, Kaixiong Tao, Peiwu Yu

**Affiliations:** 1 Department of General Surgery, Southwest Hospital, Third Military Medical University, Chongqing, 400038 China; 2 Department of Gastrointestinal Surgery, Union Hospital, Tongji Medical College, Huazhong University of Science and Technology, Wuhan, 430022 China.

## Abstract

Gli1 is a downstream transcriptional factor of Sonic hedgehog pathway in mammalians, and has been recognized as a proliferative indicator of carcinogenesis. However, its actual role in prognosis among solid malignancies remains unclear. Therefore we performed this meta-analysis aiming to discover the correlation between Gli1 positivity and clinical prognosis in patients suffering from diverse carcinomas. A total of 39 studies containing 4496 cases were selected into our quantitative analysis via electronic database search. Original data of 3-year, 5-year, 10-year overall survival and disease-free survival were extracted and calculated using odds ratio and Mantel-Haenszel model. Subgroup analysis was also conducted to clarify the possible confounding factors. P < 0.05 was considered significant in statistics. Gli1 redundancy was associated with worse 3-year, 5-year, 10-year overall survival and disease-free survival in solid malignancies. Different source regions, sample-size, mean-age and detection approaches had no impact on the negative prognostic effect of Gli1 over-expression. Nevertheless, stratified by cancer type and subcellular localization, cytoplasmic Gli1 expression and Gli1 positivity in intracranial tumors was not correlated to poorer 3-year and 5-year prognosis. The over-expression of Gli1 is a credible indicator of poorer prognosis in most of solid malignancies, irrespective of intracranial tumors.

As a life-threatening disorder and costly burden of healthcare system, cancer has drawn tremendous academic attention to the mechanisms towards its formation and uncontrollable spread. To date, accumulating evidence has disclosed the close kinship between normal embryonic development processes and malignant proliferation, both of which share similar cellular mechanisms and morphological alterations^1^. Including the conserved Sonic hedgehog (Shh) signaling, aberrant activation of pivotal developmental pathways generally facilitates carcinogenesis through a direct signal transduction in a variety of tumors, implicating a potential therapeutic targeted strategy for future medication^2^,^3^.

It is well documented that the Sonic hedgehog signaling initially emerged as an essential pathway involved in tissue growth and patterning during the fetal development^4^. The pathway has been strictly inhibited in mature tissues to ensure the physiological function and prevent abnormal proliferation. Following studies subsequently confirm its out-of-control plays a crucial role within the tumor origination and metastatic dissemination, identified as a tumor-stimulating pathway in diversified malignancies^5^. The transduction of this relatively conserved pathway is mediated by interactions between Shh, Patched and Smoothened proteins. As long as ligand Shh binds to trans-membrane protein Patched, the normally activity-inhibited Smoothened protein is then released to activate the translocation of cytoplasmic Gli1 into nucleus. Gli1 acts as a transcriptional factor downstream of the Shh pathway, whose nuclear translocation serves as a hallmark indicator of Shh pathway activation, culminating in up-regulation of target genes especially certain proliferative oncogenes^6^. Given the tumorigenic feature of Gli1, a specific targeted therapy may be prone to benefit patients with better survival quality and longer lifespan. Nevertheless, the prognostic impact of Gli1 presence in solid malignancies remains in controversy, despite an overwhelming majority of evidence has explored a negative prognostic value of Gli1 over-expression across miscellaneous neoplasms. Failed to draw a similar conclusion, Pizem *et al.*^7^ presented with a better survival status of patients under stronger nuclear Gli1 expression, suggesting an astonishing positive prognostic significance in circumstance of Gli1 redundancy. Therefore, in the present study through gathering available evidence, we carried out an exhaustive meta-analysis as well as subgroups analysis to verify the prognostic influence of Gli1 positivity across solid malignancies, aiming to provide more theoretical supports for targeted regimens.

## Results

### Eligible studies

**Figure 1:**
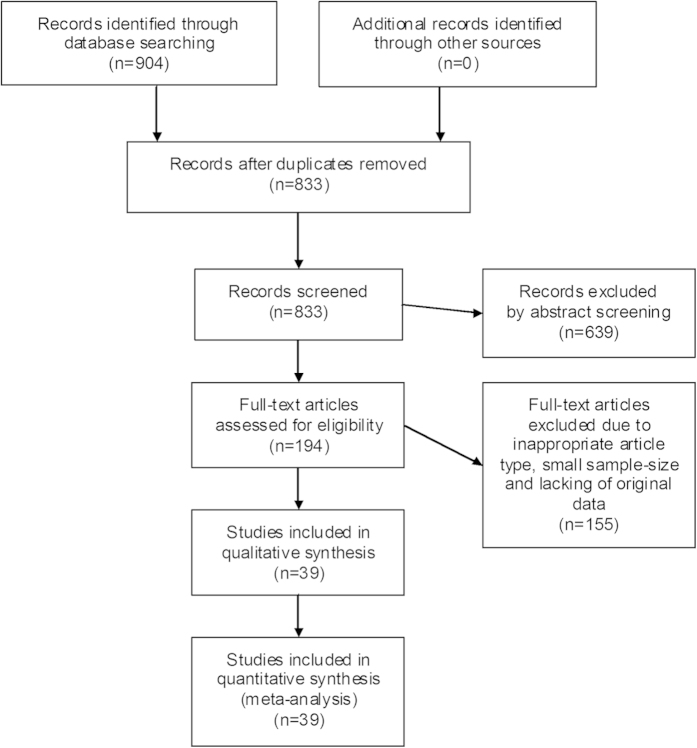
Selection flow chart of the meta-analysis.

According to the selection criteria, most of the preliminarily included entries were eliminated on account of duplicated data, inappropriate article type or inadequate original information. Eventually, a total of 39 observational studies consisting of 4496 cases were retained for subsequent pooling calculation. Figure 1 concisely displayed the selection workflow of all eligible studies in our meta-analysis.

### Demographic characteristics of included studies

As for the source regions of included studies, the majority were carried out in China (n = 21), followed by USA (n = 6), Japan (n = 5) and other sporadic nations. None of the eligible entries scored less than six by Newcastle-Ottawa Scale, revealing a high methodological quality across all studies. Studies concerning breast cancer occupied the largest proportion of cancer type among all primary literatures (n = 6), followed by hepatocellular carcinoma (n = 5), esophageal malignancy (n = 4) and remaining types of solid neoplasm. The sample-size ranged from 19 to 339, with a median of 90 patients. A total of 38 studies described the correlation of overall survival and Gli1 expression, while 11 trials reported relationship between disease-free survival and Gli1 presence. Other detailed features were recorded and summarized in Table 1.

### Correlation of Gli1 expression with 3-year overall survival and its subgroup analysis

**Figure 2:**
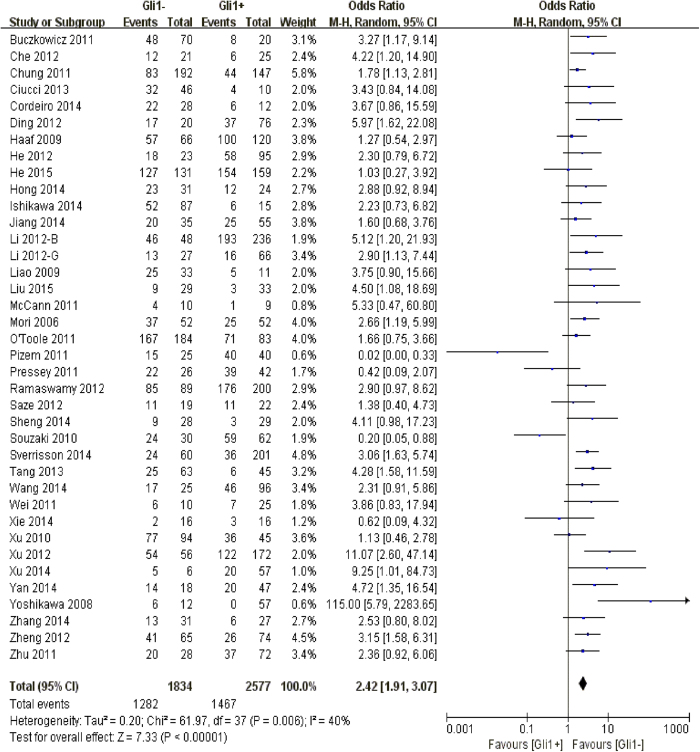
The correlation between Gli1 expression and 3-year overall survival in solid malignancies.

38 observational trials offered original data on 3-year overall survival in terms of different Gli1 expressions. It demonstrated that higher Gli1 activity referred to unfavorable 3-year overall survival (OR: 2.42, 95% CI: [1.91, 3.07], P < 0.00001). A moderate heterogeneity was observed (I^2^ = 40%) so that a random-effects model was applied for statistical adjustment. In order to explore the possible sources of heterogeneity across studies, we stratified the original articles for subgroup analysis, according to various confounding factors (Fig. 2).

In the subgroup analysis by cancer type, a worse 3-year overall survival of Gli1 positivity was observed in breast cancer (n = 6, OR: 1.63, 95% CI: [1.09, 2.45], P = 0.02, I^2^ = 1%), cancer of digestive tract (n = 8, OR: 3.51, 95% CI: [1.97, 6.24], P < 0.0001, I^2^ = 44%), liver cancer (n = 5, OR: 3.54, 95% CI: [2.23, 5.63], P < 0.00001, I^2^ = 0%), pancreatic cancer (n = 3, OR: 2.47, 95% CI: [1.24, 4.92], P = 0.01, I^2^ = 7%) and ovarian cancer (n = 3, OR: 3.80, 95% CI: [1.50, 9.61], P = 0.005, I^2^ = 0%) respectively. Nevertheless, over-expression of Gli1 in intracranial tumors was irrelevant with poorer 3-year prognosis, along with a significant heterogeneity observed (n = 5, OR: 0.99, 95% CI: [0.20, 4.98], P = 0.99, I^2^ = 84%) (Forest plot was displayed in Supplementary Figure S1).

As for different TNM clinical stages of solid malignancies, a worse 3-year survival was strongly linked to Gli1 positivity in advanced TNM stages (TNM II-IV) (n = 5, OR: 4.51, 95% CI: [1.85, 11.00], P = 0.0009, I^2^ = 35%) as well as pre-terminal stages (TNM I-III) (n = 5, OR: 4.28, 95% CI: [2.35, 7.79], P < 0.00001, I^2^ = 0%). Moreover, in studies with coverage to all stages (TNM I-IV), aberrant Gli1 activation likewise suggested a poorer 3-year overall outcome (n = 18, OR: 2.16, 95% CI: [1.55, 3.01], P < 0.00001, I^2^ = 38%) (Supplementary Figure S2).

There were totally two stratified subgroups in terms of mean age among studies. In both groups of mean age >60-year (n = 10, OR: 2.33, 95% CI: [1.52, 3.57], P < 0.0001, I^2^ = 18%) and mean age <60-year (n = 10, OR: 2.23, 95% CI: [1.35, 3.69], P = 0.002, I^2^ = 47%), negative Gli1 expression was verified to significantly associate with more favorable 3-year outcome (Supplementary Figure S3).

Subgroups analysis by Gli1 detection methods explored that high Gli1 expression status was identified as a worse prognostic marker of 3-year outcome in mRNA-examined group (n = 8, OR: 2.49, 95% CI: [1.61, 3.84], P < 0.0001, I^2^ = 7%). Similarly, obvious Gli1 positivity analyzed by immunohistochemistry (IHC) as well predicted an unfavorable 3-year overall survival in solid cancer (n = 30, OR: 2.43, 95% CI: [1.85, 3.21], P < 0.00001, I^2^ = 47%) (Supplementary Figure S4).

Included studies were divided into three subgroups according to the subcellular localization of Gli1 expression by IHC. Trials based on nuclear staining of Gli1 significantly correlated its over-reactivity with a worse 3-year survival outcome (n = 13, OR: 3.04, 95% CI: [1.82, 5.07], P < 0.0001, I^2^ = 56%), which made a similar conclusion as group of unspecific expression did (n = 14, OR: 2.27, 95% CI: [1.74, 2.96], P < 0.00001, I^2^ = 8%). However, the abnormal cytoplasmic existence of Gli1 did not stand for a detrimental tendency on 3-year overall survival through our pooling analysis (n = 3, OR: 1.11, 95% CI: [0.25, 4.83], P = 0.89, I^2^ = 76%) (Supplementary Figure S5).

Among the subgroups determined by sample size of the studies, elevated Gli1 expression was confirmed to play a worse prognostic role in terms of 3-year overall survival in solid malignancies, regardless of greater amount (>90) (n = 21, OR: 2.27, 95% CI: [1.77, 2.91], P < 0.00001, I^2^ = 35%) or smaller magnitude (<90) of participants (n = 17, OR: 2.75, 95% CI: [1.63, 4.66], P = 0.0002, I^2^ = 49%) (Supplementary Figure S6).

Stratified by source regions of the included studies, there was an analogical trend of trials implemented by Asian (n = 26, OR: 2.79, 95% CI: [2.12, 3.69], P < 0.00001, I^2^ = 30%) or non-Asian investigators (n = 12, OR: 1.83, 95% CI: [1.20, 2.78], P = 0.005, I^2^ = 50%) that Gli1 over-reactivity was identified as a poorer 3-year prognostic marker in solid tumors (Supplementary Figure S7).

### Correlation of Gli1 expression with 5-year overall survival and its subgroup analysis

**Figure 3:**
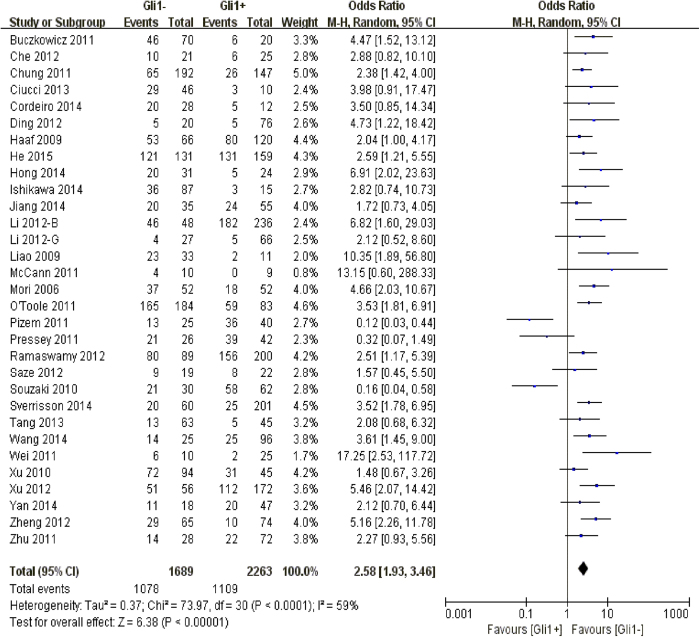
The correlation between Gli1 expression and 5-year overall survival in solid malignancies.

Concerning the 5-year overall survival in solid malignancies, the pooling analysis revealed a negative impact of Gli1 over-expression on clinical prognosis of patients, along with a moderate heterogeneity of undefined source (n = 31, OR: 2.58, 95% CI: [1.93, 3.46], P < 0.00001, I^2^ = 59%). Thus we performed the following subgroup analysis aiming to clarify the possible confounding elements (Fig. 3).

According to subgroups of different cancer types, Gli1 over-expression in breast cancer (n = 6, OR: 2.52, 95% CI: [1.83, 3.47], P < 0.00001, I^2^ = 0%), cancer of digestive tract (n = 7, OR: 3.78, 95% CI: [2.50, 5.70], P < 0.00001, I^2^ = 7%), liver cancer (n = 3, OR: 3.53, 95% CI: [1.96, 6.34], P < 0.0001, I^2^ = 0%) and ovarian cancer (n = 3, OR: 6.57, 95% CI: [2.30, 18.79], P = 0.0004, I^2^ = 0%) contributed to a significantly worse 5-year prognosis, except for intracranial tumor, in which Gli1 expression was not associated with the outcome prediction as usual (n = 5, OR: 0.93, 95% CI: [0.21, 4.19], P = 0.92, I^2^ = 87%) (Supplementary Figure S8).

With respect to subgroups by different TNM stages, our quantitative results suggested that Gli1 abundance in cancer tissues closely correlated to unfavorable 5-year overall survival in all stratified groups, including advanced stages (n = 4, OR: 2.95, 95% CI: [1.44, 6.06], P = 0.003, I^2^ = 0%), pre-terminal stages (n = 2, OR: 5.85, 95% CI: [2.61, 13.11], P < 0.0001, I^2^ = 0%) and unselected stages (n = 15, OR: 2.37, 95% CI: [1.46, 3.85], P = 0.0005, I^2^ = 66%) (Supplementary Figure S9).

Gli1 over-expression in different mean-age subgroups similarly imposed a negative effect on 5-year overall survival, especially in the group with older mean-age (Mean-age >60-year: n = 8, OR: 3.54, 95% CI: [2.19, 5.72], P < 0.00001, I^2^ = 25%) (Mean-age <60-year: n = 8, OR: 2.32, 95% CI: [1.17, 4.58], P = 0.02, I^2^ = 73%) (Supplementary Figure S10).

Different detection approaches seemed not influence the negative prognostic role of Gli1 redundancy on 5-year overall survival in solid cancer, including PCR-analyzing group (n = 8, OR: 2.85, 95% CI: [1.48, 5.49], P = 0.002, I^2^ = 48%) as well as immunohistochemistry staining group (n = 23, OR: 2.63, 95% CI: [1.88, 3.69], P < 0.00001, I^2^ = 63%) (Supplementary Figure S11).

In terms of subcellular localization of Gli1 staining, the excessive expression of Gli1 distributed in nucleus (n = 11, OR: 3.00, 95% CI: [1.61, 5.58], P = 0.0006, I^2^ = 70%) or unspecific locations (n = 9, OR: 2.61, 95% CI: [2.03, 3.36], P < 0.00001, I^2^ = 0%) referred to a disappointing 5-year overall survival in a significant way. Nevertheless, a cytoplasmic positivity of Gli1 expression failed to make a similar conclusion via the pooling analysis, displaying a dubious connection with 5-year prognosis (n = 3, OR: 1.34, 95% CI: [0.17, 10.48], P = 0.78, I^2^ = 88%) (Supplementary Figure S12).

Among the subgroups divided by different amount of sample sizes, no matter with a greater (>90) (n = 20, OR: 2.71, 95% CI: [2.07, 3.53], P < 0.00001, I^2^ = 44%) or smaller volume (<90) (n = 11, OR: 2.49, 95% CI: [1.04, 5.95], P = 0.04, I^2^ = 74%) of sample-size, the merged analysis consistently provided an unfavorable effect on 5-year overall survival owing to Gli1 aberrant activation in solid malignancies (Supplementary Figure S13).

Included studies were stratified into Asian group (n = 19, OR: 2.94, 95% CI: [2.06, 4.20], P < 0.00001, I^2^ = 50%) and non-Asian group (n = 12, OR: 2.10, 95% CI: [1.27, 3.48], P = 0.004, I^2^ = 70%) on the basis of different source regions. There was a significant association between Gli1 over-expression and poorer 5-year overall survival through our pooling analysis, regardless of the multiple nationality among the studies (Supplementary Figure S14).

### Correlation of Gli1 expression with 10-year overall survival and its subgroup analysis

**Figure 4:**
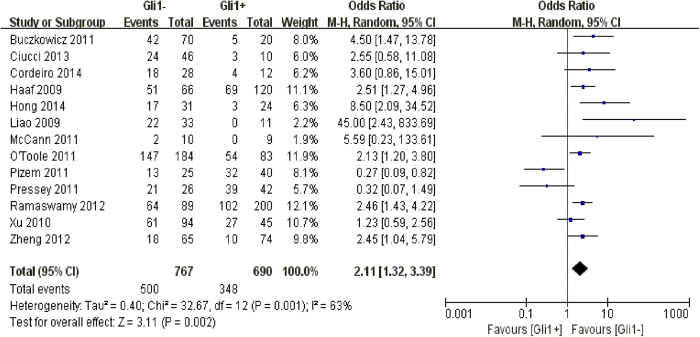
The correlation between Gli1 expression and 10-year overall survival in solid malignancies.

In our pooling analysis, Gli1 positivity was verified to have a strong connection with a worse 10-year prognosis in solid carcinomas, albeit a moderate heterogeneity was observed across studies (n = 13, OR: 2.11, 95% CI: [1.32, 3.39], P = 0.002, I^2^ = 63%). A subgroup analysis is therefore conducted (Fig. 4).

Studies were stratified according to different types of cancer. Gli1 positivity in both breast cancer (n = 4, OR: 2.10, 95% CI: [1.54, 2.86], P < 0.00001, I^2^ = 0%) and ovarian cancer (n = 3, OR: 6.28, 95% CI: [1.07, 36.69], P = 0.04, I^2^ = 38%) acted as an indicator of a worse 10-year overall survival, while its over-expression in medulloblastoma (n = 3, OR: 1.60, 95% CI: [0.25, 10.40], P = 0.62, I^2^ = 86%) was unable to offer a prognostic prediction as above (Supplementary Figure S15).

### Correlation of Gli1 expression with 3-year and 5-year disease-free survival

**Figure 5:**
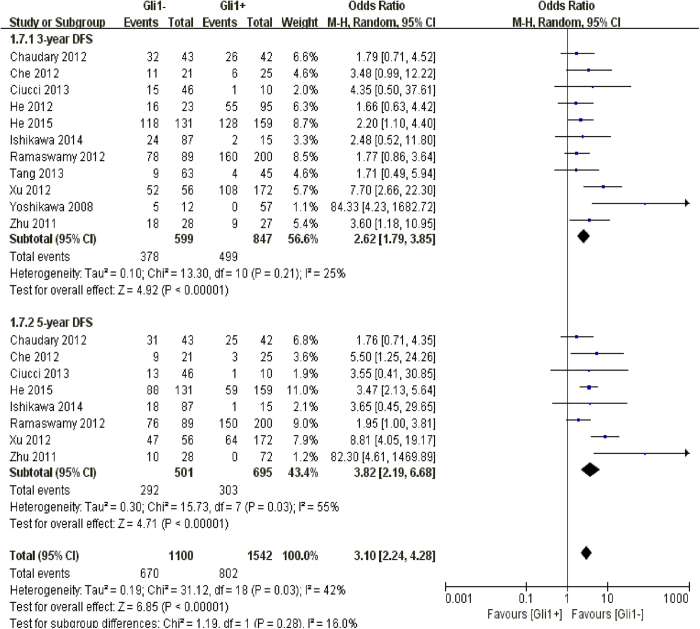
The correlation between Gli1 expression and disease-free survival in solid malignancies.

Our quantitative analysis confirmed that high Gli1 expression was shown to be an unfavorable prognostic marker of 3-year (n = 11, OR: 2.62, 95% CI: [1.79, 3.85], P < 0.00001, I^2^ = 25%) and 5-year disease-free survival (n = 8, OR: 3.82, 95% CI: [2.19, 6.68], P < 0.00001, I^2^ = 55%) among solid malignancies, along with a moderate heterogeneity existed (Fig. 5).

### Sensitivity analysis

Removal of studies featuring intracranial tumors had no substantial impact on the outcomes of 3-year (n = 33, OR: 2.52, 95% CI: [2.12, 3.00], P < 0.00001, I^2^ = 17%), 5-year (n = 26, OR: 2.95, 95% CI: [2.46, 3.54], P < 0.00001, I^2^ = 21%) and 10-year overall survival (n = 10, OR: 2.26, 95% CI: [1.47, 3.46], P = 0.0002, I^2^ = 47%), however, a huge decline on heterogeneity was observed respectively.

Exclusion of studies with mesenchymal origin obtained similar results of 3-year (n = 37, OR: 2.49, 95% CI: [1.97, 3.14], P < 0.00001, I^2^ = 37%), 5-year (n = 30, OR: 2.71, 95% CI: [2.04, 3.59], P < 0.00001, I^2^ = 56%) and 10-year overall survival (n = 12, OR: 2.34, 95% CI: [1.49, 3.67], P = 0.0002, I^2^ = 59%) respectively.

Elimination of studies scoring 6 in NOS assessment was unable to alter the negative prognostic effect of Gli1 positivity in terms of 3-year (n = 30, OR: 2.30, 95% CI: [1.71, 3.08], P < 0.00001, I^2^ = 49%), 5-year (n = 25, OR: 2.29, 95% CI: [1.63, 3.22], P < 0.00001, I^2^ = 62%) and 10-year overall survival among solid malignancies (n = 11, OR: 1.95, 95% CI: [1.12, 3.38], P = 0.02, I^2^ = 67%).

### Publication bias

**Figure 6:**
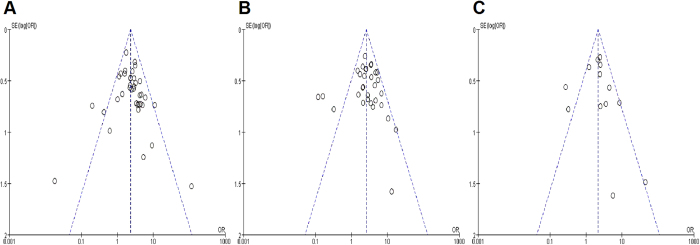
The funnel plots of this meta-analysis. (**A**) 3-year overall survival; (**B**) 5-year overall survival; (**C**) 10-year overall survival.

The funnel plots, Egger test and Begg test jointly demonstrated that there was no publication bias concerning 3-year (Egger: P = 0.505; Begg: P = 0.138), 5-year (Egger: P = 0.996; Begg: P = 0.415) and 10-year (Egger: P = 0.653; Begg: P = 0.583) overall survival in our meta-analysis (Fig. 6).

## Discussion

As the laboratorial evidence has revealed, inactivation of Sonic hedgehog pathway is generally marked by translocation of Gli1 transcriptional factor into the nucleus. The frequently over-expressed Gli1 strongly triggers the carcinogenesis and dissemination in various cancer models. Its oncogenic role takes effects by up-regulation of the downstream target genes, especially including certain detrimental oncoproteins^8^. The pivotal action of Gli1 inside the molecular carcinogenesis network emphasizes its potential value for targeted treatment modalities. However, from the clinical perspective, a persuasive support of Gli1’s clinical significance is still unavailable, partially due to the uncertainty of the association between Gli1 positivity and prognosis implication. The majority of investigations established potent evidence suggesting an unfavorable impact of Gli1 abnormality on clinical prognosis in a wide spectrum of carcinomas. Nevertheless, on the contrary, several researchers recently highlighted that an obvious advantage on survival duration was obtained in Gli1 over-expression cases, with mechanisms not fully elucidated^7^. A comprehensive study is therefore in urgent demand.

To our knowledge, the present study is the first and most full-scale meta-analysis systemically exploring the possible prognostic role of Gli1 up-regulation in solid malignancies. On the whole, our quantitative results strongly supported the current mainstream viewpoint that an undesirable impact of Gli1 redundancy was correlated with the 3-year, 5-year, 10-year overall survival and disease-free survival, taking no account of subgroup confounding factors. Additionally, this negative prognostic role was confirmed to be independent of mean-age, source countries, detection measures, sample-size and TNM stages. With respect to subcellular localization, nuclear emergence of Gli1 was identified to tightly associate with awful prognosis while cytoplasmic expression culminated in an absolutely contrary conclusion that there was no obvious connection between Gli1 cytoplasmic expression and worse prognosis in solid malignancies. This inconsistency is probably attributed to the academic consensus that translocation of Gli1 into the nucleus is the hallmark of inactivation of Sonic hedgehog pathway, exclusive of the cytoplasmic positioning pattern^9^. Moreover, Gli1 lost its prediction efficacy in intracranial tumors by subgroup analysis, displaying an indistinctive prognostic effect on long-term survival. The possible explanation of this exception may be owing to the less possibility of metastasis and cerebrospinal fluid dissemination in medulloblastomas with Gli1 nuclear staining, although the molecular mechanism remains unclear^10^. Furthermore, Gershon *et al.* reported a proliferation-inhibitory effect of Gli1 over-expression on neuroblastoma cell lines, which triggered the malignancy towards mature differentiation instead^11^. In spite of aforementioned plausible reasons, it warrants more investigations to make further explanations to these controversies.

Apart from the inspiring outcomes, limitations still lay in this quantitative meta-analysis. First of all, despite the usage of random-effects model and subgroup analysis, the heterogeneity across studies failed to be eliminated completely, which could result in bias of the outcome in certain extent. Secondly, on account of the lacking of effective data, we merely analyzed the correlation between Gli1 redundancy and prognosis in terms of certain clinical elements. Other parameters that may partially contribute to the heterogeneity were not explored, such as pathological grade and body mass index.

In spite of the limitations mentioned above, there are still numerous valuable implications of this comprehensive mete-analysis. Firstly, the formerly recognized oncoprotein Gli1 has been identified as a biomarker for poor prognosis of 3-year, 5-year, 10-year overall survival and disease-free survival in solid malignancies for the first time, irrespective of several exceptions including intracranial tumors. Secondly, nuclear localization instead of cytoplasmic positivity serves as a prognostic predictor in most cancers, which provides a more specific subcellular positioning to guide the clinical evaluations for diagnosis and prognosis.

## Methods

### Search strategy

We performed a thorough search for available literatures in electronic databases of PubMed and Web of Science until September 2015. The search terms “Gli1 AND (cancer OR neoplasm OR carcinoma OR malignancy)” was applied and we initially identified 904 entries for further examination. Both abstracts and full-texts were elaborately screened to exclude irrelevant articles. Citation lists of retrieved articles were additionally reviewed to guarantee the sensitivity of the search process. Two authors independently carried out this procedure and any discrepancy was resolved by mutual discussion.

### Selection criteria

Studies that met the following requirements were considered eligible and selected into our quantitative meta-analysis: 1. English-written articles published from 2000–2015; 2. Studies exploring the correlation between Gli1 expression and prognosis in human solid malignancies; 3. A minimal follow-up duration of 3 years.

Studies were eliminated on the basis of the following criteria: 1. Duplicated or overlapped studies; 2. Studies with a sample-size less than 10 participants; 3. Lack of enough statistical data for further quantification calculation; 4. Review articles or case reports.

All evaluations were separately undertaken by two individuals to warrant the precision of selection process.

### Data extraction

By aid of predefined standardized extraction forms, two investigators independently extracted data from each qualified studies, in terms of general information, 3-year survival, 5-year survival, 10-year survival and disease-free survival respectively. The original survival data were obtained from the text, tables or Kaplan-Meier curves for both comparative groups. GetData Graph Digitizer 2.2 helped us to digitize and extract survival information from the Kaplan-Meier curves. A joint decision was offered in the case of any disagreement.

### Methodological quality assessment

Given that all of the included studies were observational studies, a Newcastle-Ottawa Scale (NOS) was adopted to assist the quality assessment of each eligible article. The scale was revised with certain adaptive modifications to match the practical needs of the pooled analysis. There were generally three categories contained in the scale, including selection, comparability and outcome, with a maximum score of nine. Studies graded with more than six scores were classified as high quality literatures in methodology. Two reviewers independently conducted the assessment process and a consensus on NOS score of each study had achieved.

### Statistical analysis

Review Manager 5.3 was adopted for the quantitative calculation in this meta-analysis. The original data was inserted as dichotomous variables, therefore odds ratio (OR) along with 95% confidential interval (CI) was applied to measure the correlation between Gli1 presence and long-term survival, including the general survival analysis and sub-groups comparison. I^2^ was designated as an indicator of heterogeneity across studies, with its value below 25% defined as low heterogeneity. In the absence of significant heterogeneity (I^2^ < 25%), a fixed-effects model was appropriately employed, while a random-effects model took its place in the remaining situations. P < 0.05 certainly signified a statistical significance within the comparisons. Additionally, we implemented a sensitivity analysis examining the consistency of the pooled outcomes. Funnel plots as well as Egger’s test and Begg’s test was used to investigate the internal publication bias across the included studies, based on the calculation by Stata 12.0 software.

## Additional Information

**How to cite this article**: Cheng, J. *et al.* Prognostic role of Gli1 expression in solid malignancies: a meta-analysis. *Sci. Rep.* **6**, 22184; doi: 10.1038/srep22184 (2016).

## Supplementary Material

Supplementary Information

## Figures and Tables

**Table 1:** Demographic information of included studies.

Reference	Country	Cancer type	No.	Mean age	Male/Female	TNM stage	Follow-up (range) months	Gli1(−/+) No.	3-year OS(−/+)%	5-year OS(−/+)%	10-year OS(−/+)%	NOS score
Li *et al.*^12^	China	Gallbladder carcinoma	93	NA	39/54	I–IV	32(5–66)	27/66	48.1/24.2	14.8/7.6	NA	7
Xie *et al.*^9^	China	Gallbladder carcinoma	32	67.0 ± 12.0	11/21	I–IV	20	16/16	18.8/12.5	NA	NA	7
Hong *et al.*^13^	China	Lung cancer	55	61.9 ± 8.1	34/21	I–IV	NA	31/24	74.2/50.0	64.5/20.8	54.8/12.5	8
Ishikawa *et al.*^14^	Japan	Lung cancer	102	64.8 ± 9.8	68/34	II–IV	NA	87/15	59.8/40.0	41.4/20.0	NA	7
Che *et al.*^15^	China	Liver cancer	46	51.7 ± 11.2	40/6	NA	30(1–83)	21/25	57.1/24.0	47.6/24.0	NA	7
Zheng *et al.*^16^	USA	Liver cancer	139	NA	NA	NA	NA	65/74	63.1/35.1	44.6/13.5	27.7/13.5	6
Tang *et al.*^17^	China	Liver cancer	108	NA	77/31	I–IV	17(2–82)	63/45	39.7/13.3	20.6/11.1	NA	6
Xu *et al.*^18^	China	Liver cancer	63	NA	49/14	I–IV	NA	6/57	83.3/35.1	NA	NA	7
Zhang *et al.*^19^	China	Liver cancer	58	51.7	43/15	I–IV	23(3–36)	31/27	41.9/22.2	NA	NA	6
Chaudary *et al.*^20^	Canada	Cervical cancer	85	47.0	All female	I–IV	72(9–127)	43/42	NA	NA	NA	7
Pressey *et al.*^21^	USA	Rhabdomyosarcoma	68	NA	46/22	I–IV	70(0-123)	26/42	84.6/92.9	80.8/92.9	80.8/92.9	7
Yan *et al.*^22^	China	Glioma	65	NA	18/47	II–IV	>3 years	18/47	77.8/42.6	61.1/42.6	NA	7
Ding *et al.*^23^	China	Colon cancer	96	NA	60/36	I–IV	38(6–60)	20/76	85.0/48.7	25.0/6.6	NA	7
Xu *et al.*^24^	China	Colon cancer	228	NA	108/120	I–III	52(5–109)	56/172	96.4/70.9	91.1/65.1	NA	7
Liao *et al.*^25^	China	Ovarian cancer	44	NA	All female	I–IV	64(5–111)	33/11	75.8/45.5	69.7/18.2	66.7/0.0	7
McCann *et al.*^26^	USA	Ovarian cancer	19	61.0 ± 13.6	All female	III–IV	NA	10/9	40.0/11.1	40.0/0.0	20.0/0.0	8
Ciucci *et al.*^27^	Italy	Ovarian cancer	56	54.0	All female	III–IV	35(9–127)	46/10	69.6/40.0	63.0/30.0	52.2/30.0	8
He *et al.*^28^	China	Bladder cancer	118	56.0	105/13	I–III	NA	23/95	78.3/61.1	NA	NA	7
Sverrisson *et al.*^29^	China	Bladder cancer	261	NA	194/67	I–IV	33	60/201	40.0/17.9	33.3/12.4	NA	7
Haaf *et al.*^30^	Germany	Breast cancer	186	56.0	All female	NA	78(0–148)	66/120	86.4/83.3	80.3/66.7	77.3/57.5	8
Xu *et al.*^31^	USA	Breast cancer	139	NA	All female	I–IV	94	94/45	81.9/80.0	76.6/68.9	64.9/60.0	7
O’Toole *et al.*^32^	USA	Breast cancer	267	55.0	All female	NA	NA	184/83	90.8/85.5	89.7/71.1	79.9/65.1	7
Li *et al.*^33^	China	Breast cancer	284	52.0	All female	I–III	62(3–83)	48/236	95.8/81.8	95.8/77.1	NA	7
Ramaswamy *et al.*^34^	USA	Breast cancer	289	54.5	All female	NA	96(1–139)	89/200	95.5/88.0	89.9/78.0	71.9/51.0	8
He *et al.*^35^	China	Breast cancer	290	NA	All female	NA	NA	131/159	96.9/96.6	92.4/82.4	NA	6
Souzaki *et al.*^36^	Japan	Neuroblastoma	92	NA	56/36	I–IV	NA	30/62	80.0/95.2	70.0/93.5	NA	7
Mori *et al.*^8^	Japan	Esophageal cancer	104	63.0	92/12	I–IV	NA	52/52	71.2/48.1	71.2/34.6	NA	6
Yoshikawa *et al.*^37^	Japan	Esophageal cancer	69	60.7	58/11	II–IV	NA	12/57	50.0/0.0	NA	NA	7
Zhu *et al.*^38^	China	Esophageal cancer	100	NA	85/15	I–IV	23(3–83)	28/72	71.4/51.4	50.0/30.6	NA	7
Wei *et al.*^39^	China	Esophageal cancer	35	60.0	29/6	I–IV	NA	10/25	60.0/28.0	60.0/8.0	NA	6
Pizem *et al.*^40^	Slovenia	Medulloblastoma	65	12.4	51/14	NA	71(2–234)	25/40	60.0/100.0	52.0/90.0	52.0/80.0	8
Buczkowicz *et al.*^41^	Canada	Medulloblastoma	90	6.6	NA	NA	80	70/20	68.6/40.0	65.7/30.0	60.0/25.0	6
Cordeiro *et al.*^42^	Brazil	Medulloblastoma	40	NA	26/14	NA	NA	28/12	78.6/50.0	71.4/41.7	64.3/33.3	7
Chung *et al.*^43^	USA	Head and neck squamous cell carcinoma	339	NA	263/76	NA	96	192/147	43.2/29.9	33.9/17.7	NA	7
Saze *et al.*^44^	Japan	Gastric cancer	41	64.8	30/11	I–IV	NA	19/22	57.9/50.0	47.4/36.4	NA	8
Wang *et al.*^45^	China	Gastric cancer	121	63.0	92/29	I–IV	30	25/96	68.8/47.9	56.0/26.0	NA	8
Jiang *et al.*^46^	China	Pancreatic cancer	90	62.0	57/33	I–IV	31(0–87)	35/55	57.1/45.5	57.1/43.6	NA	7
Sheng *et al.*^47^	China	Pancreatic cancer	57	NA	38/19	I–III	NA	28/29	32.1/10.3	NA	NA	6
Liu *et al.*^48^	China	Pancreatic cancer	62	NA	43/19	I–III	NA	29/33	31.0/9.1	NA	NA	7

NOS: Newcastle-Ottawa Scale; NA: not available.

## References

[b1] Hanahan D. & Weinberg R. A. Hallmarks of cancer: the next generation. Cell 144, 646–674 (2011). 21376230 10.1016/j.cell.2011.02.013

[b2] Omenetti A. & Diehl A. M. The adventures of sonic hedgehog in development and repair. II. Sonic hedgehog and liver development, inflammation, and cancer. Am J Physiol Gastrointest Liver Physiol 294, G595–G598 (2008). 18218671 10.1152/ajpgi.00543.2007

[b3] Hui M. *et al.* The Hedgehog signalling pathway in breast development, carcinogenesis and cancer therapy. Breast Cancer Res 15, 203 (2013). 23547970 10.1186/bcr3401 PMC3672663

[b4] Ingham P. W. & McMahon A. P. Hedgehog signaling in animal development: paradigms and principles. Genes Dev 15, 3059–3087 (2001). 11731473 10.1101/gad.938601

[b5] Pasca D. M. M. & Hebrok M. Hedgehog signalling in cancer formation and maintenance. Nat Rev Cancer 3, 903–911 (2003). 14737121 10.1038/nrc1229

[b6] Ma X. *et al.* Frequent activation of the hedgehog pathway in advanced gastric adenocarcinomas. Carcinogenesis 26, 1698–1705 (2005). 15905200 10.1093/carcin/bgi130

[b7] Pizem J., Popovic M. & Cor A. Expression of Gli1 and PARP1 in medulloblastoma: an immunohistochemical study of 65 cases. J Neurooncol 103, 459–467 (2011). 20953661 10.1007/s11060-010-0431-2

[b8] Mori Y., Okumura T., Tsunoda S., Sakai Y. & Shimada Y. Gli-1 expression is associated with lymph node metastasis and tumor progression in esophageal squamous cell carcinoma. Oncology-Basel 70, 378–389 (2006). 10.1159/000098111 17179732

[b9] Xie F. *et al.* Aberrant activation of Sonic hedgehog signaling in chronic cholecystitis and gallbladder carcinoma. Hum Pathol 45, 513–521 (2014). 24440094 10.1016/j.humpath.2013.10.017

[b10] Kool M. *et al.* Integrated genomics identifies five medulloblastoma subtypes with distinct genetic profiles, pathway signatures and clinicopathological features. PLoS One 3, e3088 (2008). 18769486 10.1371/journal.pone.0003088 PMC2518524

[b11] Gershon T. R. *et al.* Enteric neural crest differentiation in ganglioneuromas implicates Hedgehog signaling in peripheral neuroblastic tumor pathogenesis. PLoS One 4, e7491 (2009). 19834598 10.1371/journal.pone.0007491 PMC2759000

[b12] Li J. *et al.* Immunohistochemical evidence of the prognostic value of hedgehog pathway components in primary gallbladder carcinoma. Surg Today 42, 770–775 (2012). 22407314 10.1007/s00595-012-0157-1

[b13] Hong Z. *et al.* Activation of hedgehog signaling pathway in human non-small cell lung cancers. Pathol Oncol Res 20, 917–922 (2014). 24710823 10.1007/s12253-014-9774-x

[b14] Ishikawa M. *et al.* Expression of the GLI family genes is associated with tumor progression in advanced lung adenocarcinoma. World J Surg Oncol 12, 253 (2014). 25103784 10.1186/1477-7819-12-253 PMC4249769

[b15] Che L., Yuan Y. H., Jia J. & Ren J. Activation of sonic hedgehog signaling pathway is an independent potential prognosis predictor in human hepatocellular carcinoma patients. Chin J Cancer Res 24, 323–331 (2012). 23359030 10.3978/j.issn.1000-9604.2012.10.10 PMC3551326

[b16] Zheng X. *et al.* The transcription factor GLI1 mediates TGFbeta1 driven EMT in hepatocellular carcinoma via a SNAI1-dependent mechanism. PLoS One 7, e49581 (2012). 23185371 10.1371/journal.pone.0049581 PMC3501480

[b17] Tang L. *et al.* The prognostic significance and therapeutic potential of hedgehog signaling in intrahepatic cholangiocellular carcinoma. Clin Cancer Res 19, 2014–2024 (2013). 23493353 10.1158/1078-0432.CCR-12-0349

[b18] Xu Q. *et al.* The transcriptional activity of Gli1 is negatively regulated by AMPK through Hedgehog partial agonism in hepatocellular carcinoma. Int J Mol Med 34, 733–741 (2014). 25017332 10.3892/ijmm.2014.1847 PMC4121351

[b19] Zhang J. *et al.* Evaluation of Jagged2 and Gli1 expression and their correlation with prognosis in human hepatocellular carcinoma. Mol Med Rep 10, 749–754 (2014). 24858621 10.3892/mmr.2014.2246

[b20] Chaudary N. *et al.* Hedgehog pathway signaling in cervical carcinoma and outcome after chemoradiation. Cancer 118, 3105–3115 (2012). 22028038 10.1002/cncr.26635

[b21] Pressey J. G., Anderson J. R., Crossman D. K., Lynch J. C. & Barr F. G. Hedgehog pathway activity in pediatric embryonal rhabdomyosarcoma and undifferentiated sarcoma: a report from the Children’s Oncology Group. Pediatr Blood Cancer 57, 930–938 (2011). 21618411 10.1002/pbc.23174 PMC3164386

[b22] Yan G. N. *et al.* Endothelial cells promote stem-like phenotype of glioma cells through activating the Hedgehog pathway. J Pathol 234, 11–22 (2014). 24604164 10.1002/path.4349 PMC4260128

[b23] Ding Y. L., Zhou Y., Xiang L., Ji Z. P. & Luo Z. H. Expression of glioma-associated oncogene homolog 1 is associated with invasion and postoperative liver metastasis in colon cancer. Int J Med Sci 9, 334–338 (2012). 22745574 10.7150/ijms.4553 PMC3384915

[b24] Xu M., Li X., Liu T., Leng A. & Zhang G. Prognostic value of hedgehog signaling pathway in patients with colon cancer. Med Oncol 29, 1010–1016 (2012). 21424326 10.1007/s12032-011-9899-7

[b25] Liao X. *et al.* Aberrant activation of hedgehog signaling pathway in ovarian cancers: effect on prognosis, cell invasion and differentiation. Carcinogenesis 30, 131–140 (2009). 19028702 10.1093/carcin/bgn230 PMC7109814

[b26] McCann C. K. *et al.* Inhibition of Hedgehog signaling antagonizes serous ovarian cancer growth in a primary xenograft model. PLoS One 6, e28077 (2011). 22140510 10.1371/journal.pone.0028077 PMC3226669

[b27] Ciucci A. *et al.* Expression of the glioma-associated oncogene homolog 1 (gli1) in advanced serous ovarian cancer is associated with unfavorable overall survival. PLoS One 8, e60145 (2013). 23555905 10.1371/journal.pone.0060145 PMC3610749

[b28] He H. C. *et al.* Expression of hedgehog pathway components is associated with bladder cancer progression and clinical outcome. Pathol Oncol Res 18, 349–355 (2012). 21861243 10.1007/s12253-011-9451-2

[b29] Sverrisson E. F. *et al.* Clinicopathological correlates of Gli1 expression in a population-based cohort of patients with newly diagnosed bladder cancer. Urol Oncol 32, 539–545 (2014). 24856810 10.1016/j.urolonc.2014.03.006 PMC4243987

[b30] Ten H. A. *et al.* Expression of the glioma-associated oncogene homolog (GLI) 1 in human breast cancer is associated with unfavourable overall survival. Bmc Cancer 9, 298 (2009). 19706168 10.1186/1471-2407-9-298 PMC2753634

[b31] Xu L. *et al.* Gli1 promotes cell survival and is predictive of a poor outcome in ERalpha-negative breast cancer. Breast Cancer Res Treat 123, 59–71 (2010). 19902354 10.1007/s10549-009-0617-5 PMC2888711

[b32] O’Toole S. A. *et al.* Hedgehog overexpression is associated with stromal interactions and predicts for poor outcome in breast cancer. Cancer Res 71, 4002–4014 (2011). 21632555 10.1158/0008-5472.CAN-10-3738

[b33] Li Y. H. *et al.* Overexpression of Gli1 in cancer interstitial tissues predicts early relapse after radical operation of breast cancer. Chin J Cancer Res 24, 263–274 (2012). 23358704 10.3978/j.issn.1000-9604.2012.10.04 PMC3551322

[b34] Ramaswamy B. *et al.* Hedgehog signaling is a novel therapeutic target in tamoxifen-resistant breast cancer aberrantly activated by PI3K/AKT pathway. Cancer Res 72, 5048–5059 (2012). 22875023 10.1158/0008-5472.CAN-12-1248 PMC3837449

[b35] He M. *et al.* The Hedgehog signalling pathway mediates drug response of MCF-7 mammosphere cells in breast cancer patients. Clin Sci (Lond) 129, 809–822 (2015). 26201092 10.1042/CS20140592

[b36] Souzaki R. *et al.* Hedgehog signaling pathway in neuroblastoma differentiation. J Pediatr Surg 45, 2299–2304 (2010). 21129534 10.1016/j.jpedsurg.2010.08.020

[b37] Yoshikawa R. *et al.* Hedgehog signal activation in oesophageal cancer patients undergoing neoadjuvant chemoradiotherapy. Br J Cancer 98, 1670–1674 (2008). 18475300 10.1038/sj.bjc.6604361 PMC2391133

[b38] Zhu W. *et al.* Correlation of hedgehog signal activation with chemoradiotherapy sensitivity and survival in esophageal squamous cell carcinomas. Jpn J Clin Oncol 41, 386–393 (2011). 21127038 10.1093/jjco/hyq217

[b39] Wei L. & Xu Z. Cross-signaling among phosphinositide-3 kinase, mitogen-activated protein kinase and sonic hedgehog pathways exists in esophageal cancer. Int J Cancer 129, 275–284 (2011). 20839260 10.1002/ijc.25673

[b40] Pizem J., Popovic M. & Cor A. Expression of Gli1 and PARP1 in medulloblastoma: an immunohistochemical study of 65 cases. J Neurooncol 103, 459–467 (2011). 20953661 10.1007/s11060-010-0431-2

[b41] Buczkowicz P., Ma J. & Hawkins C. GLI2 is a potential therapeutic target in pediatric medulloblastoma. J Neuropathol Exp Neurol 70, 430–437 (2011). 21572341 10.1097/NEN.0b013e31821b94db

[b42] Cordeiro B. M. *et al.* SHH, WNT, and NOTCH pathways in medulloblastoma: when cancer stem cells maintain self-renewal and differentiation properties. Childs Nerv Syst 30, 1165–1172 (2014). 24695855 10.1007/s00381-014-2403-x

[b43] Chung C. H. *et al.* Glioma-associated oncogene family zinc finger 1 expression and metastasis in patients with head and neck squamous cell carcinoma treated with radiation therapy (RTOG 9003). J Clin Oncol 29, 1326–1334 (2011). 21357786 10.1200/JCO.2010.32.3295 PMC3084000

[b44] Saze Z. *et al.* Activation of the sonic hedgehog pathway and its prognostic impact in patients with gastric cancer. Dig Surg 29, 115–123 (2012). 22456124 10.1159/000336949

[b45] Wang Z. S. *et al.* Significance and prognostic value of Gli-1 and Snail/E-cadherin expression in progressive gastric cancer. Tumour Biol 35, 1357–1363 (2014). 24081672 10.1007/s13277-013-1185-1

[b46] Jiang H. *et al.* Expression of Gli1 and Wnt2B correlates with progression and clinical outcome of pancreatic cancer. Int J Clin Exp Pathol 7, 4531–4538 (2014). 25120849 PMC4129084

[b47] Sheng W. *et al.* The clinicopathological significance and relationship of Gli1, MDM2 and p53 expression in resectable pancreatic cancer. Histopathology 64, 523–535 (2014). 24289472 10.1111/his.12273

[b48] Liu Q. *et al.* Gli1 promotes transforming growth factor-beta1- and epidermal growth factor-induced epithelial to mesenchymal transition in pancreatic cancer cells. Surgery 158, 211–224 (2015). 25979438 10.1016/j.surg.2015.03.016

