# Corrigendum: Prognostic role of Gli1 expression in solid malignancies: a meta-analysis

**DOI:** 10.1038/srep27166

**Published:** 2016-06-02

**Authors:** Ji Cheng, Jinbo Gao, Kaixiong Tao

Scientific Reports 6: Article number: 22184;10.1038/srep22184 Published online: 02222016; Updated: 06022016

Peiwu Yu did not contribute directly to this study and has been removed from the author list.

The Acknowledgements section now reads:

“We sincerely appreciate Peiwu Yu for providing linguistic support. This study has been financially supported by Research Fund of Public Welfare in Health Industry, Health and Family Plan Committee of China (No. 201402015) and National Natural Science Foundation of China (No. 81572413).”

The Author Contributions statement now reads:

“J.C. and K.T. contributed to the design of the study and manuscript writing. J.C. and J.G. contributed to the data extraction and analysis process of the study. K.T. contributed to the financial support and revision of the manuscript.”

In addition, Ji Cheng was incorrectly listed as being affiliated with ‘Department of General Surgery, Southwest Hospital, Third Military Medical University, Chongqing, 400038 China’. The correct affiliation is listed below:

Department of Gastrointestinal Surgery, Union Hospital, Tongji Medical College, Huazhong University of Science and Technology, Wuhan, 430022 China.

These errors have now been corrected in the PDF and HTML versions of the Article, as well as the Supplementary Information file.

