# Supplementary material for: Prognostic role of Gli1 expression in solid malignancies: a meta-analysis
Source: Sci Rep. 2016 Feb 22;6:22184. doi: 10.1038/srep22184 (PMC4762019; doi:10.1038/srep22184)
Supplement: Supplementary Information [file srep22184-s1.pdf]

Prognostic role of Gli1 expression in solid malignancies: a meta-analysis

Ji Cheng[1], Jinbo Gao[2], Peiwu Yu[1]*, Kaixiong Tao[2]*

Figure S1

Study or Subgroup	Gli1- Events	Gli1- Total	Gli1+ Events	Gli1+ Total	Weight	Odds Ratio M-H, Random, 95% CI
**1.2.1 Breast cancer**						
Haaf 2009	57	66	100	120	4.8%	1.27 [0.54, 2.97]
He 2015	127	131	154	159	3.1%	1.03 [0.27, 3.92]
Li 2012-B	46	48	193	236	2.7%	5.12 [1.20, 21.93]
O'Toole 2011	167	184	71	83	5.1%	1.66 [0.75, 3.66]
Ramaswamy 2012	85	89	176	200	3.8%	2.90 [0.97, 8.62]
Xu 2010	77	94	36	45	4.6%	1.13 [0.46, 2.78]
**Subtotal (95% CI)**		**612**		**843**	**24.2%**	**1.63 [1.09, 2.45]**
Total events	559		730			
Heterogeneity: Tau² = 0.00; Chi² = 5.03, df = 5 (P = 0.41); I² = 1%						
Test for overall effect: Z = 2.36 (P = 0.02)						
**1.2.2 Cancer of digestive tract**						
Ding 2012	17	20	37	76	3.1%	5.97 [1.62, 22.08]
Mori 2006	37	52	25	52	5.0%	2.66 [1.19, 5.99]
Saze 2012	11	19	11	22	3.3%	1.38 [0.40, 4.73]
Wang 2014	17	25	46	96	4.5%	2.31 [0.91, 5.86]
Wei 2011	6	10	7	25	2.5%	3.86 [0.83, 17.94]
Xu 2012	54	56	122	172	2.7%	11.07 [2.60, 47.14]
Yoshikawa 2008	6	12	0	57	0.9%	115.00 [5.79, 2283.65]
Zhu 2011	20	28	37	72	4.4%	2.36 [0.92, 6.06]
**Subtotal (95% CI)**		**222**		**572**	**26.6%**	**3.51 [1.97, 6.24]**
Total events	168		285			
Heterogeneity: Tau² = 0.29; Chi² = 12.59, df = 7 (P = 0.08); I² = 44%						
Test for overall effect: Z = 4.27 (P < 0.0001)						
**1.2.3 Intracranial tumor**						
Buczkowicz 2011	48	70	8	20	4.1%	3.27 [1.17, 9.14]
Cordeiro 2014	22	28	6	12	2.8%	3.67 [0.86, 15.59]
Pizem 2011	15	25	40	40	0.9%	0.02 [0.00, 0.33]
Souzaki 2010	24	30	59	62	2.7%	0.20 [0.05, 0.88]
Yan 2014	14	18	20	47	3.3%	4.72 [1.35, 16.54]
**Subtotal (95% CI)**		**171**		**181**	**13.8%**	**0.99 [0.20, 4.98]**
Total events	123		133			
Heterogeneity: Tau² = 2.70; Chi² = 24.58, df = 4 (P < 0.0001); I² = 84%						
Test for overall effect: Z = 0.01 (P = 0.99)						
**1.2.4 Liver cancer**						
Che 2012	12	21	6	25	3.3%	4.22 [1.20, 14.90]
Tang 2013	25	63	6	45	4.2%	4.28 [1.58, 11.59]
Xu 2014	5	6	20	57	1.5%	9.25 [1.01, 84.73]
Zhang 2014	13	31	6	27	3.6%	2.53 [0.80, 8.02]
Zheng 2012	41	65	26	74	5.6%	3.15 [1.58, 6.31]
**Subtotal (95% CI)**		**186**		**228**	**18.1%**	**3.54 [2.23, 5.63]**
Total events	96		64			
Heterogeneity: Tau² = 0.00; Chi² = 1.37, df = 4 (P = 0.85); I² = 0%						
Test for overall effect: Z = 5.36 (P < 0.00001)						
**1.2.5 Pancreatic cancer**						
Jiang 2014	20	35	25	55	4.8%	1.60 [0.68, 3.76]
Liu 2015	9	29	3	33	2.8%	4.50 [1.08, 18.69]
Sheng 2014	9	28	3	29	2.8%	4.11 [0.98, 17.23]
**Subtotal (95% CI)**		**92**		**117**	**10.4%**	**2.47 [1.24, 4.92]**
Total events	38		31			
Heterogeneity: Tau² = 0.03; Chi² = 2.16, df = 2 (P = 0.34); I² = 7%						
Test for overall effect: Z = 2.57 (P = 0.01)						
**1.2.6 Ovarian cancer**						
Ciucci 2013	32	46	4	10	2.8%	3.43 [0.84, 14.08]
Liao 2009	25	33	5	11	2.8%	3.75 [0.90, 15.66]
McCann 2011	4	10	1	9	1.3%	5.33 [0.47, 60.80]
**Subtotal (95% CI)**		**89**		**30**	**6.9%**	**3.80 [1.50, 9.61]**
Total events	61		10			
Heterogeneity: Tau² = 0.00; Chi² = 0.10, df = 2 (P = 0.95); I² = 0%						
Test for overall effect: Z = 2.82 (P = 0.005)						
**Total (95% CI)**		**1372**		**1971**	**100.0%**	**2.60 [1.93, 3.49]**
Total events	1045		1253			
Heterogeneity: Tau² = 0.28; Chi² = 52.97, df = 29 (P = 0.004); I² = 45%						
Test for overall effect: Z = 6.31 (P < 0.00001)						
Test for subgroup differences: Chi² = 9.78, df = 5 (P = 0.08), I² = 48.9%						

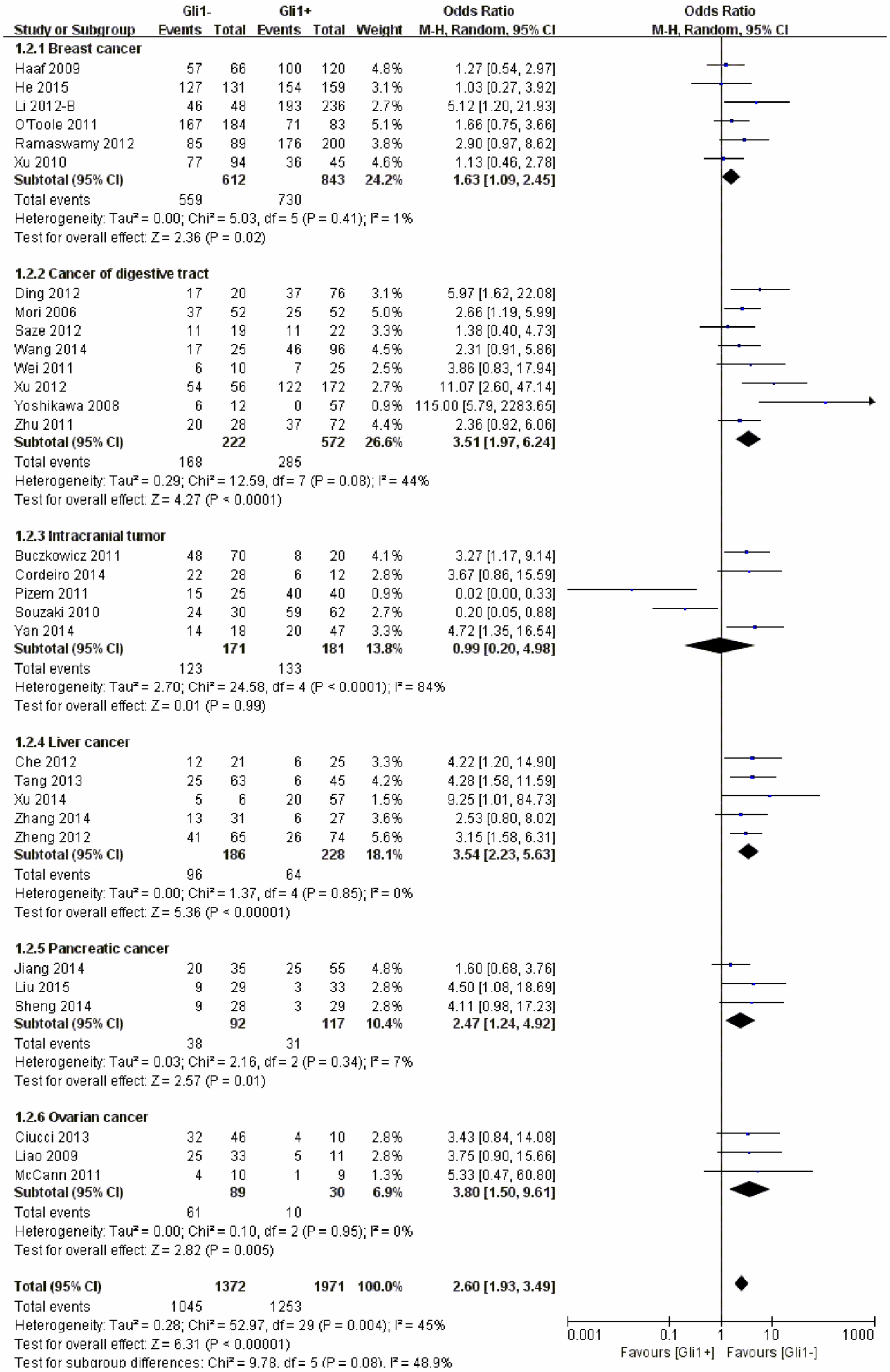

Figure S2

Study or Subgroup	Gli1- Events	Gli1- Total	Gli1+ Events	Gli1+ Total	Weight	Odds Ratio M-H, Random, 95% CI
**1.8.1 Advanced stages (TNM II-IV)**						
Ciucci 2013	32	46	4	10	3.0%	3.43 [0.84, 14.08]
Ishikawa 2014	52	87	6	15	4.0%	2.23 [0.73, 6.82]
McCann 2011	4	10	1	9	1.2%	5.33 [0.47, 60.80]
Yan 2014	14	18	20	47	3.5%	4.72 [1.35, 16.54]
Yoshikawa 2008	6	12	0	57	0.8%	115.00 [5.79, 2283.65]
**Subtotal (95% CI)**		**173**		**138**	**12.6%**	**4.51 [1.85, 11.00]**
Total events	108		31			
Heterogeneity: Tau² = 0.35; Chi² = 6.19, df = 4 (P = 0.19); I² = 35%						
Test for overall effect: Z = 3.32 (P = 0.0009)						
**1.8.2 Pre-terminal stages (TNM I-III)**						
He 2012	18	23	58	95	4.3%	2.30 [0.79, 6.72]
Li 2012-B	46	48	193	236	2.8%	5.12 [1.20, 21.93]
Liu 2015	9	29	3	33	2.9%	4.50 [1.08, 18.69]
Sheng 2014	9	28	3	29	2.9%	4.11 [0.98, 17.23]
Xu 2012	54	56	122	172	2.9%	11.07 [2.60, 47.14]
**Subtotal (95% CI)**		**184**		**565**	**15.8%**	**4.28 [2.35, 7.79]**
Total events	136		379			
Heterogeneity: Tau² = 0.00; Chi² = 3.15, df = 4 (P = 0.53); I² = 0%						
Test for overall effect: Z = 4.77 (P < 0.00001)						
**1.8.5 All stages (TNM I-IV)**						
Ding 2012	17	20	37	76	3.3%	5.97 [1.62, 22.08]
Hong 2014	23	31	12	24	4.0%	2.88 [0.92, 8.94]
Jiang 2014	20	35	25	55	5.4%	1.60 [0.68, 3.76]
Li 2012-G	13	27	16	66	4.9%	2.90 [1.13, 7.44]
Liao 2009	25	33	5	11	2.9%	3.75 [0.90, 15.66]
Mori 2006	37	52	25	52	5.7%	2.66 [1.19, 5.99]
Pressey 2011	22	26	39	42	2.5%	0.42 [0.09, 2.07]
Saze 2012	11	19	11	22	3.6%	1.38 [0.40, 4.73]
Souzaki 2010	24	30	59	62	2.8%	0.20 [0.05, 0.88]
Sverrisson 2014	24	60	36	201	7.0%	3.06 [1.63, 5.74]
Tang 2013	25	63	6	45	4.6%	4.28 [1.58, 11.59]
Wang 2014	17	25	46	96	5.0%	2.31 [0.91, 5.86]
Wei 2011	6	10	7	25	2.6%	3.86 [0.83, 17.94]
Xie 2014	2	16	3	16	1.8%	0.62 [0.09, 4.32]
Xu 2010	77	94	36	45	5.2%	1.13 [0.46, 2.78]
Xu 2014	5	6	20	57	1.4%	9.25 [1.01, 84.73]
Zhang 2014	13	31	6	27	3.9%	2.53 [0.80, 8.02]
Zhu 2011	20	28	37	72	4.9%	2.36 [0.92, 6.06]
**Subtotal (95% CI)**		**606**		**994**	**71.7%**	**2.16 [1.55, 3.01]**
Total events	381		426			
Heterogeneity: Tau² = 0.19; Chi² = 27.62, df = 17 (P = 0.05); I² = 38%						
Test for overall effect: Z = 4.53 (P < 0.00001)						
**Total (95% CI)**		**963**		**1697**	**100.0%**	**2.64 [1.98, 3.51]**
Total events	625		836			
Heterogeneity: Tau² = 0.20; Chi² = 42.54, df = 27 (P = 0.03); I² = 37%						
Test for overall effect: Z = 6.63 (P < 0.00001)						
Test for subgroup differences: Chi² = 5.30, df = 2 (P = 0.07), I² = 62.3%						

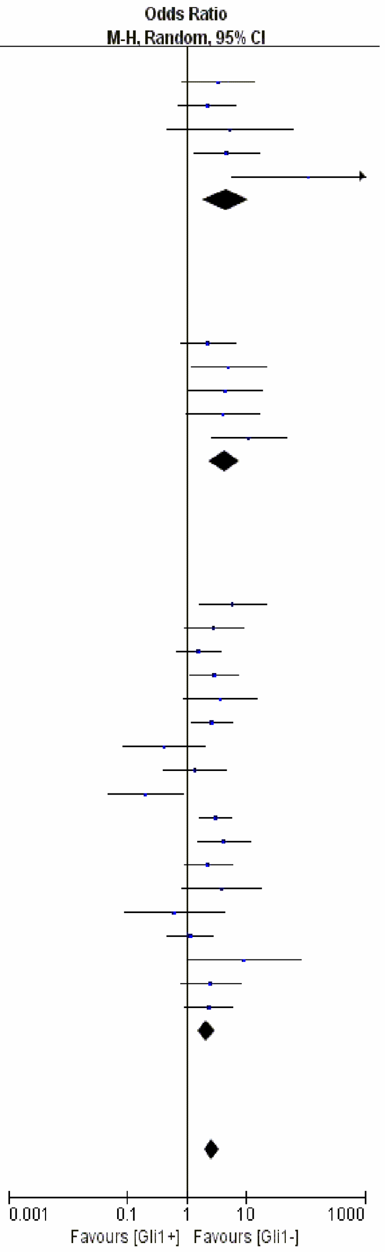

Figure S3

Study or Subgroup	Gli1- Events	Gli1- Total	Gli1+ Events	Gli1+ Total	Weight	Odds Ratio M-H, Random, 95% CI
**1.9.1 Mean age > 60**						
Hong 2014	23	31	12	24	5.4%	2.88 [0.92, 8.94]
Ishikawa 2014	52	87	6	15	5.5%	2.23 [0.73, 6.82]
Jiang 2014	20	35	25	55	7.7%	1.60 [0.68, 3.76]
McCann 2011	4	10	1	9	1.6%	5.33 [0.47, 60.80]
Mori 2006	37	52	25	52	8.2%	2.66 [1.19, 5.99]
Saze 2012	11	19	11	22	4.8%	1.38 [0.40, 4.73]
Wang 2014	17	25	46	96	7.0%	2.31 [0.91, 5.86]
Wei 2011	6	10	7	25	3.4%	3.86 [0.83, 17.94]
Xie 2014	2	16	3	16	2.3%	0.62 [0.09, 4.32]
Yoshikawa 2008	6	12	0	57	1.1%	115.00 [5.79, 2283.65]
**Subtotal (95% CI)**		**297**		**371**	**47.2%**	**2.33 [1.52, 3.57]**
Total events	178		136			
Heterogeneity: Tau² = 0.08; Chi² = 10.95, df = 9 (P = 0.28); I² = 18%						
Test for overall effect: Z = 3.90 (P < 0.0001)						
**1.9.2 Mean age < 60**						
Buczkowicz 2011	48	70	8	20	6.2%	3.27 [1.17, 9.14]
Che 2012	12	21	6	25	4.7%	4.22 [1.20, 14.90]
Ciucci 2013	32	46	4	10	3.9%	3.43 [0.84, 14.08]
Haaf 2009	57	66	100	120	7.8%	1.27 [0.54, 2.97]
He 2012	18	23	58	95	5.9%	2.30 [0.79, 6.72]
Li 2012-B	46	48	193	236	3.8%	5.12 [1.20, 21.93]
O'Toole 2011	167	184	71	83	8.4%	1.66 [0.75, 3.66]
Pizem 2011	15	25	40	40	1.1%	0.02 [0.00, 0.33]
Ramaswamy 2012	85	89	176	200	5.7%	2.90 [0.97, 8.62]
Zhang 2014	13	31	6	27	5.3%	2.53 [0.80, 8.02]
**Subtotal (95% CI)**		**603**		**856**	**52.8%**	**2.23 [1.35, 3.69]**
Total events	493		662			
Heterogeneity: Tau² = 0.29; Chi² = 16.91, df = 9 (P = 0.05); I² = 47%						
Test for overall effect: Z = 3.14 (P = 0.002)						
**Total (95% CI)**		**900**		**1227**	**100.0%**	**2.29 [1.67, 3.16]**
Total events	671		798			
Heterogeneity: Tau² = 0.15; Chi² = 27.33, df = 19 (P = 0.10); I² = 30%						
Test for overall effect: Z = 5.11 (P < 0.00001)						
Test for subgroup differences: Chi² = 0.02, df = 1 (P = 0.90), I² = 0%						

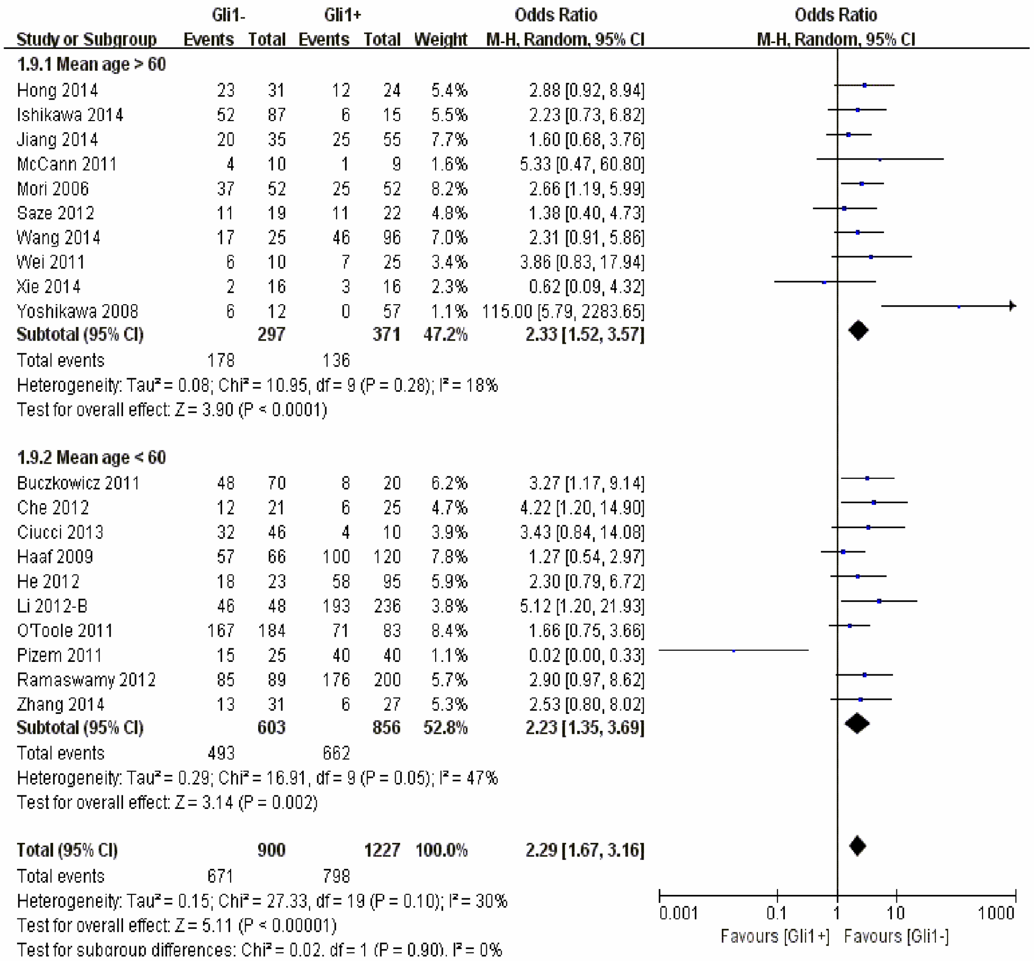

Figure S4

Study or Subgroup	Gli1- Events	Gli1- Total	Gli1+ Events	Gli1+ Total	Weight	Odds Ratio M-H, Random, 95% CI
**1.11.1 PCR**						
Che 2012	12	21	6	25	2.4%	4.22 [1.20, 14.90]
Cordeiro 2014	22	28	6	12	2.0%	3.67 [0.86, 15.59]
Hong 2014	23	31	12	24	2.7%	2.88 [0.92, 8.94]
Ishikawa 2014	52	87	6	15	2.8%	2.23 [0.73, 6.82]
McCann 2011	4	10	1	9	0.8%	5.33 [0.47, 60.80]
Pressey 2011	22	26	39	42	1.7%	0.42 [0.09, 2.07]
Saze 2012	11	19	11	22	2.4%	1.38 [0.40, 4.73]
Zheng 2012	41	65	26	74	4.5%	3.15 [1.58, 6.31]
**Subtotal (95% CI)**		**287**		**223**	**19.4%**	**2.49 [1.61, 3.84]**
Total events	187		107			
Heterogeneity: Tau² = 0.03; Chi² = 7.56, df = 7 (P = 0.37); I² = 7%						
Test for overall effect: Z = 4.12 (P < 0.0001)						
**1.11.2 Immunohistochemistry (IHC)**						
Buczkowicz 2011	48	70	8	20	3.1%	3.27 [1.17, 9.14]
Chung 2011	83	192	44	147	5.8%	1.78 [1.13, 2.81]
Ciucci 2013	32	46	4	10	2.0%	3.43 [0.84, 14.08]
Ding 2012	17	20	37	76	2.3%	5.97 [1.62, 22.08]
Haaf 2009	57	66	100	120	3.8%	1.27 [0.54, 2.97]
He 2012	18	23	58	95	2.9%	2.30 [0.79, 6.72]
He 2015	127	131	154	159	2.2%	1.03 [0.27, 3.92]
Jiang 2014	20	35	25	55	3.8%	1.60 [0.68, 3.76]
Li 2012-B	46	48	193	236	1.9%	5.12 [1.20, 21.93]
Li 2012-G	13	27	16	66	3.4%	2.90 [1.13, 7.44]
Liao 2009	25	33	5	11	2.0%	3.75 [0.90, 15.66]
Liu 2015	9	29	3	33	2.0%	4.50 [1.08, 18.69]
Mori 2006	37	52	25	52	4.0%	2.66 [1.19, 5.99]
O'Toole 2011	167	184	71	83	4.0%	1.66 [0.75, 3.66]
Pizem 2011	15	25	40	40	0.6%	0.02 [0.00, 0.33]
Ramaswamy 2012	85	89	176	200	2.9%	2.90 [0.97, 8.62]
Sheng 2014	9	28	3	29	2.0%	4.11 [0.98, 17.23]
Souzaki 2010	24	30	59	62	1.9%	0.20 [0.05, 0.88]
Sverrisson 2014	24	60	36	201	4.8%	3.06 [1.63, 5.74]
Tang 2013	25	63	6	45	3.2%	4.28 [1.58, 11.59]
Wang 2014	17	25	46	96	3.4%	2.31 [0.91, 5.86]
Wei 2011	6	10	7	25	1.8%	3.86 [0.83, 17.94]
Xie 2014	2	16	3	16	1.2%	0.62 [0.09, 4.32]
Xu 2010	77	94	36	45	3.6%	1.13 [0.46, 2.78]
Xu 2012	54	56	122	172	2.0%	11.07 [2.60, 47.14]
Xu 2014	5	6	20	57	1.0%	9.25 [1.01, 84.73]
Yan 2014	14	18	20	47	2.4%	4.72 [1.35, 16.54]
Yoshikawa 2008	6	12	0	57	0.6%	115.00 [5.79, 2283.65]
Zhang 2014	13	31	6	27	2.7%	2.53 [0.80, 8.02]
Zhu 2011	20	28	37	72	3.4%	2.36 [0.92, 6.06]
**Subtotal (95% CI)**		**1547**		**2354**	**80.6%**	**2.43 [1.85, 3.21]**
Total events	1095		1360			
Heterogeneity: Tau² = 0.25; Chi² = 54.27, df = 29 (P = 0.003); I² = 47%						
Test for overall effect: Z = 6.30 (P < 0.00001)						
**Total (95% CI)**		**1834**		**2577**	**100.0%**	**2.42 [1.91, 3.07]**
Total events	1282		1467			
Heterogeneity: Tau² = 0.20; Chi² = 61.97, df = 37 (P = 0.006); I² = 40%						
Test for overall effect: Z = 7.33 (P < 0.00001)						
Test for subgroup differences: Chi² = 0.01, df = 1 (P = 0.94), I² = 0%						

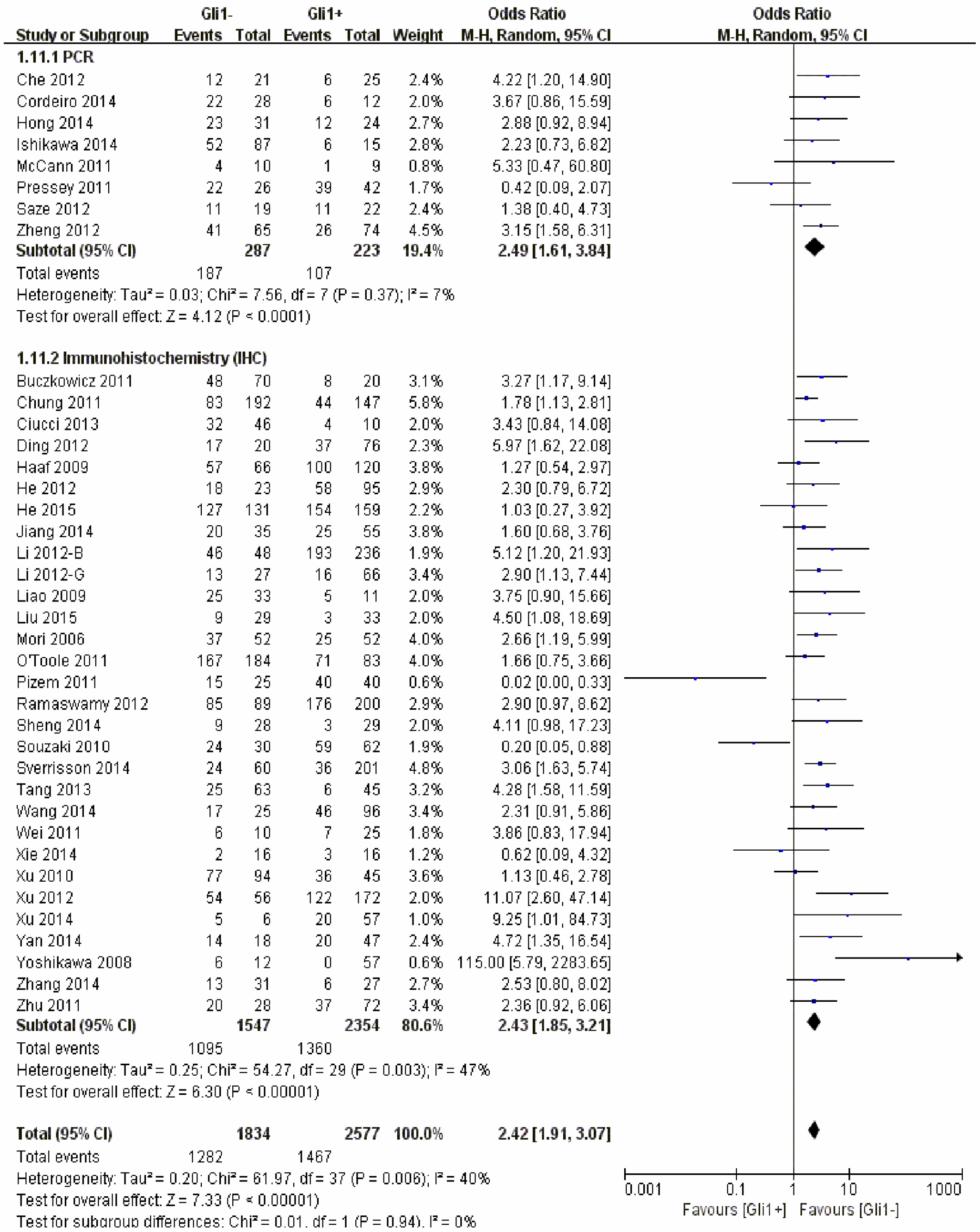

Figure S5

Study or Subgroup	Gli1- Events	Gli1- Total	Gli1+ Events	Gli1+ Total	Weight	Odds Ratio M-H, Random, 95% CI
**1.13.1 Cytoplasmic expression**						
Jiang 2014	20	35	25	55	4.6%	1.60 [0.68, 3.76]
Liao 2009	25	33	5	11	2.6%	3.75 [0.90, 15.66]
Souzaki 2010	24	30	59	62	2.5%	0.20 [0.05, 0.88]
**Subtotal (95% CI)**		**98**		**128**	**9.6%**	**1.11 [0.25, 4.83]**
Total events	69		89			
Heterogeneity: Tau² = 1.28; Chi² = 8.51, df = 2 (P = 0.01); I² = 76%						
Test for overall effect: Z = 0.14 (P = 0.89)						
**1.13.2 Nuclear expression**						
Buczkowicz 2011	48	70	8	20	3.8%	3.27 [1.17, 9.14]
Ciucci 2013	32	46	4	10	2.6%	3.43 [0.84, 14.08]
Ding 2012	17	20	37	76	2.9%	5.97 [1.62, 22.08]
Haaf 2009	57	66	100	120	4.6%	1.27 [0.54, 2.97]
He 2012	18	23	58	95	3.7%	2.30 [0.79, 6.72]
Li 2012-B	46	48	193	236	2.5%	5.12 [1.20, 21.93]
Li 2012-G	13	27	16	66	4.2%	2.90 [1.13, 7.44]
Mori 2006	37	52	25	52	4.8%	2.66 [1.19, 5.99]
Pizem 2011	15	25	40	40	0.8%	0.02 [0.00, 0.33]
Wei 2011	6	10	7	25	2.3%	3.86 [0.83, 17.94]
Xu 2012	54	56	122	172	2.5%	11.07 [2.60, 47.14]
Yoshikawa 2008	6	12	0	57	0.8%	115.00 [5.79, 2283.65]
Zhu 2011	20	28	37	72	4.2%	2.36 [0.92, 6.06]
**Subtotal (95% CI)**		**483**		**1041**	**39.7%**	**3.04 [1.82, 5.07]**
Total events	369		647			
Heterogeneity: Tau² = 0.45; Chi² = 27.05, df = 12 (P = 0.008); I² = 56%						
Test for overall effect: Z = 4.25 (P < 0.0001)						
**1.13.3 Unspecific expression**						
Chung 2011	83	192	44	147	6.7%	1.78 [1.13, 2.81]
He 2015	127	131	154	159	2.8%	1.03 [0.27, 3.92]
Liu 2015	9	29	3	33	2.6%	4.50 [1.08, 18.69]
O'Toole 2011	167	184	71	83	4.9%	1.66 [0.75, 3.66]
Ramaswamy 2012	85	89	176	200	3.6%	2.90 [0.97, 8.62]
Sheng 2014	9	28	3	29	2.6%	4.11 [0.98, 17.23]
Sverrisson 2014	24	60	36	201	5.7%	3.06 [1.63, 5.74]
Tang 2013	25	63	6	45	4.0%	4.28 [1.58, 11.59]
Wang 2014	17	25	46	96	4.2%	2.31 [0.91, 5.86]
Xie 2014	2	16	3	16	1.6%	0.62 [0.09, 4.32]
Xu 2010	77	94	36	45	4.4%	1.13 [0.46, 2.78]
Xu 2014	5	6	20	57	1.3%	9.25 [1.01, 84.73]
Yan 2014	14	18	20	47	3.0%	4.72 [1.35, 16.54]
Zhang 2014	13	31	6	27	3.4%	2.53 [0.80, 8.02]
**Subtotal (95% CI)**		**966**		**1185**	**50.7%**	**2.27 [1.74, 2.96]**
Total events	657		624			
Heterogeneity: Tau² = 0.02; Chi² = 14.09, df = 13 (P = 0.37); I² = 8%						
Test for overall effect: Z = 6.06 (P < 0.00001)						
**Total (95% CI)**		**1547**		**2354**	**100.0%**	**2.43 [1.85, 3.21]**
Total events	1095		1360			
Heterogeneity: Tau² = 0.25; Chi² = 54.27, df = 29 (P = 0.003); I² = 47%						
Test for overall effect: Z = 6.30 (P < 0.00001)						
Test for subgroup differences: Chi² = 2.03, df = 2 (P = 0.36), I² = 1.7%						

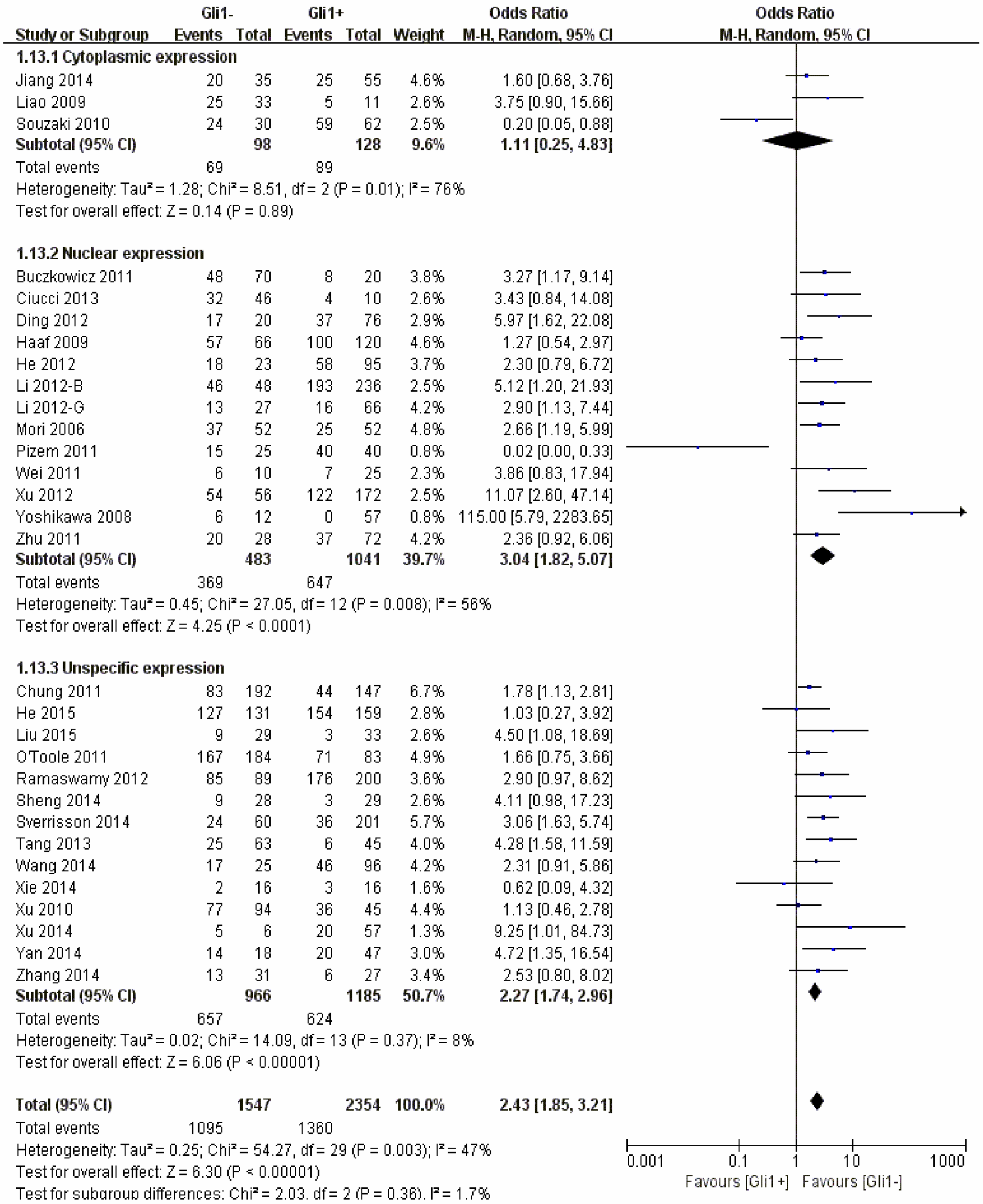

Figure S6

Study or Subgroup	Gli1- Events	Gli1- Total	Gli1+ Events	Gli1+ Total	Weight	Odds Ratio M-H, Random, 95% CI
**1.16.1 Sample-size > 90**						
Buczkowicz 2011	48	70	8	20	3.1%	3.27 [1.17, 9.14]
Chung 2011	83	192	44	147	5.8%	1.78 [1.13, 2.81]
Ding 2012	17	20	37	76	2.3%	5.97 [1.62, 22.08]
Haaf 2009	57	66	100	120	3.8%	1.27 [0.54, 2.97]
He 2012	18	23	58	95	2.9%	2.30 [0.79, 6.72]
He 2015	127	131	154	159	2.2%	1.03 [0.27, 3.92]
Ishikawa 2014	52	87	6	15	2.8%	2.23 [0.73, 6.82]
Jiang 2014	20	35	25	55	3.8%	1.60 [0.68, 3.76]
Li 2012-B	46	48	193	236	1.9%	5.12 [1.20, 21.93]
Li 2012-G	13	27	16	66	3.4%	2.90 [1.13, 7.44]
Mori 2006	37	52	25	52	4.0%	2.66 [1.19, 5.99]
O'Toole 2011	167	184	71	83	4.0%	1.66 [0.75, 3.66]
Ramaswamy 2012	85	89	176	200	2.9%	2.90 [0.97, 8.62]
Souzaki 2010	24	30	59	62	1.9%	0.20 [0.05, 0.88]
Sverrisson 2014	24	60	36	201	4.8%	3.06 [1.63, 5.74]
Tang 2013	25	63	6	45	3.2%	4.28 [1.58, 11.59]
Wang 2014	17	25	46	96	3.4%	2.31 [0.91, 5.86]
Xu 2010	77	94	36	45	3.6%	1.13 [0.46, 2.78]
Xu 2012	54	56	122	172	2.0%	11.07 [2.60, 47.14]
Zheng 2012	41	65	26	74	4.5%	3.15 [1.58, 6.31]
Zhu 2011	20	28	37	72	3.4%	2.36 [0.92, 6.06]
**Subtotal (95% CI)**		**1445**		**2091**	**69.6%**	**2.27 [1.77, 2.91]**
Total events	1052		1281			
Heterogeneity: Tau² = 0.11; Chi² = 30.59, df = 20 (P = 0.06); I² = 35%						
Test for overall effect: Z = 6.42 (P < 0.00001)						
**1.16.2 Sample-size < 90**						
Che 2012	12	21	6	25	2.4%	4.22 [1.20, 14.90]
Ciucci 2013	32	46	4	10	2.0%	3.43 [0.84, 14.08]
Cordeiro 2014	22	28	6	12	2.0%	3.67 [0.86, 15.59]
Hong 2014	23	31	12	24	2.7%	2.88 [0.92, 8.94]
Liao 2009	25	33	5	11	2.0%	3.75 [0.90, 15.66]
Liu 2015	9	29	3	33	2.0%	4.50 [1.08, 18.69]
McCann 2011	4	10	1	9	0.8%	5.33 [0.47, 60.80]
Pizem 2011	15	25	40	40	0.6%	0.02 [0.00, 0.33]
Pressey 2011	22	26	39	42	1.7%	0.42 [0.09, 2.07]
Saze 2012	11	19	11	22	2.4%	1.38 [0.40, 4.73]
Sheng 2014	9	28	3	29	2.0%	4.11 [0.98, 17.23]
Wei 2011	6	10	7	25	1.8%	3.86 [0.83, 17.94]
Xie 2014	2	16	3	16	1.2%	0.62 [0.09, 4.32]
Xu 2014	5	6	20	57	1.0%	9.25 [1.01, 84.73]
Yan 2014	14	18	20	47	2.4%	4.72 [1.35, 16.54]
Yoshikawa 2008	6	12	0	57	0.6%	115.00 [5.79, 2283.65]
Zhang 2014	13	31	6	27	2.7%	2.53 [0.80, 8.02]
**Subtotal (95% CI)**		**389**		**486**	**30.4%**	**2.75 [1.63, 4.66]**
Total events	230		186			
Heterogeneity: Tau² = 0.57; Chi² = 31.31, df = 16 (P = 0.01); I² = 49%						
Test for overall effect: Z = 3.77 (P = 0.0002)						
**Total (95% CI)**		**1834**		**2577**	**100.0%**	**2.42 [1.91, 3.07]**
Total events	1282		1467			
Heterogeneity: Tau² = 0.20; Chi² = 61.97, df = 37 (P = 0.006); I² = 40%						
Test for overall effect: Z = 7.33 (P < 0.00001)						
Test for subgroup differences: Chi² = 0.42, df = 1 (P = 0.52), I² = 0%						

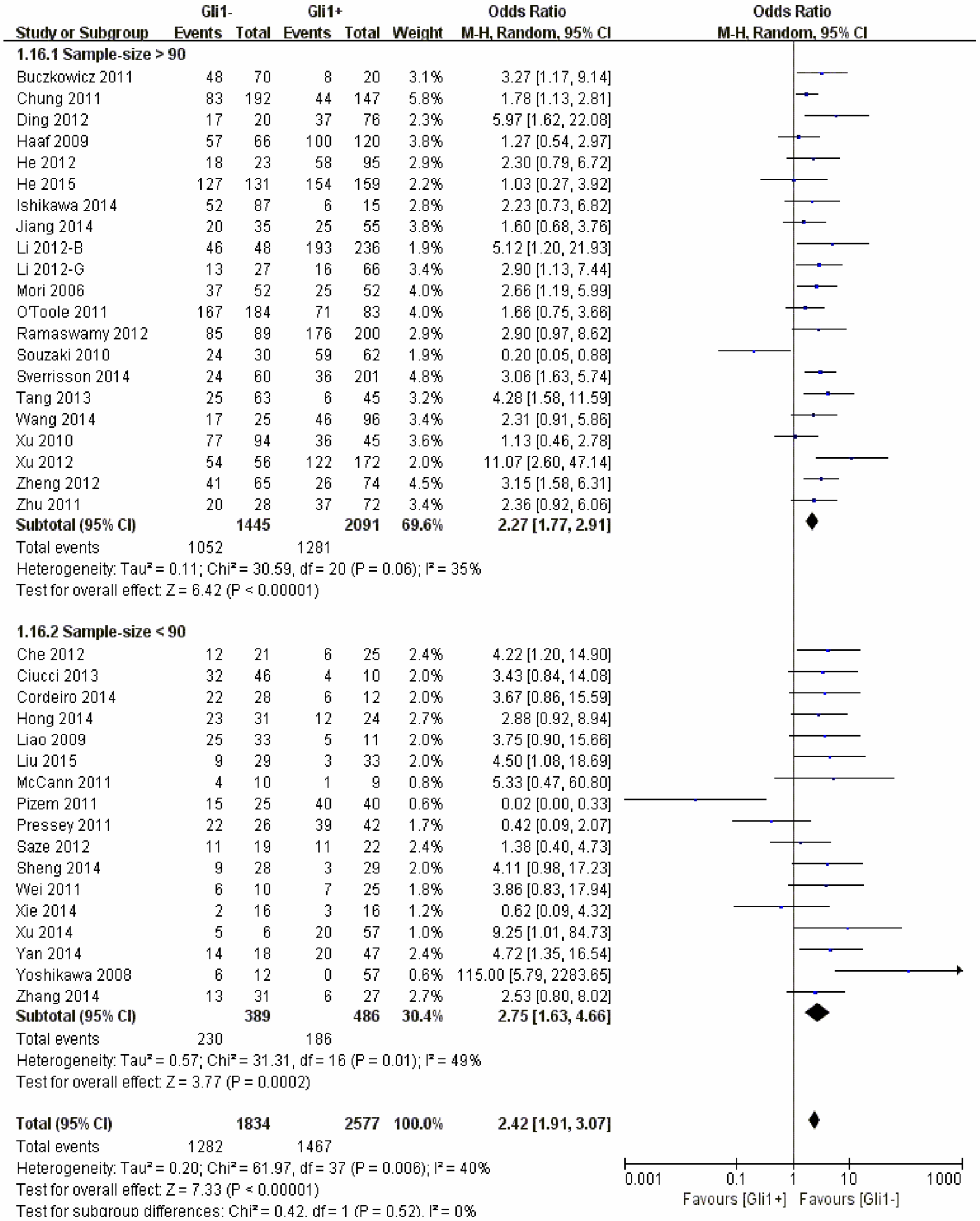

Figure S7

Study or Subgroup	Gli1- Events	Gli1- Total	Gli1+ Events	Gli1+ Total	Weight	Odds Ratio M-H, Random, 95% CI
**1.18.1 Asian studies**						
Che 2012	12	21	6	25	2.4%	4.22 [1.20, 14.90]
Ding 2012	17	20	37	76	2.3%	5.97 [1.62, 22.08]
He 2012	18	23	58	95	2.9%	2.30 [0.79, 6.72]
He 2015	127	131	154	159	2.2%	1.03 [0.27, 3.92]
Hong 2014	23	31	12	24	2.7%	2.88 [0.92, 8.94]
Ishikawa 2014	52	87	6	15	2.8%	2.23 [0.73, 6.82]
Jiang 2014	20	35	25	55	3.8%	1.60 [0.68, 3.76]
Li 2012-B	46	48	193	236	1.9%	5.12 [1.20, 21.93]
Li 2012-G	13	27	16	66	3.4%	2.90 [1.13, 7.44]
Liao 2009	25	33	5	11	2.0%	3.75 [0.90, 15.66]
Liu 2015	9	29	3	33	2.0%	4.50 [1.08, 18.69]
Mori 2006	37	52	25	52	4.0%	2.66 [1.19, 5.99]
Saze 2012	11	19	11	22	2.4%	1.38 [0.40, 4.73]
Sheng 2014	9	28	3	29	2.0%	4.11 [0.98, 17.23]
Souzaki 2010	24	30	59	62	1.9%	0.20 [0.05, 0.88]
Sverrisson 2014	24	60	36	201	4.8%	3.06 [1.63, 5.74]
Tang 2013	25	63	6	45	3.2%	4.28 [1.58, 11.59]
Wang 2014	17	25	46	96	3.4%	2.31 [0.91, 5.86]
Wei 2011	6	10	7	25	1.8%	3.86 [0.83, 17.94]
Xie 2014	2	16	3	16	1.2%	0.62 [0.09, 4.32]
Xu 2012	54	56	122	172	2.0%	11.07 [2.60, 47.14]
Xu 2014	5	6	20	57	1.0%	9.25 [1.01, 84.73]
Yan 2014	14	18	20	47	2.4%	4.72 [1.35, 16.54]
Yoshikawa 2008	6	12	0	57	0.6%	115.00 [5.79, 2283.65]
Zhang 2014	13	31	6	27	2.7%	2.53 [0.80, 8.02]
Zhu 2011	20	28	37	72	3.4%	2.36 [0.92, 6.06]
**Subtotal (95% CI)**		**939**		**1775**	**65.2%**	**2.79 [2.12, 3.69]**
Total events	629		916			
Heterogeneity: $Tau^2 = 0.15$; $Chi^2 = 35.86$, df = 25 (P = 0.07); $I^2 = 30\%$						
Test for overall effect: Z = 7.23 (P < 0.00001)						
**1.18.2 Non-Asian studies**						
Buczkowicz 2011	48	70	8	20	3.1%	3.27 [1.17, 9.14]
Chung 2011	83	192	44	147	5.8%	1.78 [1.13, 2.81]
Ciucci 2013	32	46	4	10	2.0%	3.43 [0.84, 14.08]
Cordeiro 2014	22	28	6	12	2.0%	3.67 [0.86, 15.59]
Haaf 2009	57	66	100	120	3.8%	1.27 [0.54, 2.97]
McCann 2011	4	10	1	9	0.8%	5.33 [0.47, 60.80]
O'Toole 2011	167	184	71	83	4.0%	1.66 [0.75, 3.66]
Pizem 2011	15	25	40	40	0.6%	0.02 [0.00, 0.33]
Pressey 2011	22	26	39	42	1.7%	0.42 [0.09, 2.07]
Ramaswamy 2012	85	89	176	200	2.9%	2.90 [0.97, 8.62]
Xu 2010	77	94	36	45	3.6%	1.13 [0.46, 2.78]
Zheng 2012	41	65	26	74	4.5%	3.15 [1.58, 6.31]
**Subtotal (95% CI)**		**895**		**802**	**34.8%**	**1.83 [1.20, 2.78]**
Total events	653		551			
Heterogeneity: $Tau^2 = 0.24$; $Chi^2 = 22.08$, df = 11 (P = 0.02); $I^2 = 50\%$						
Test for overall effect: Z = 2.81 (P = 0.005)						
**Total (95% CI)**		**1834**		**2577**	**100.0%**	**2.42 [1.91, 3.07]**
Total events	1282		1467			
Heterogeneity: $Tau^2 = 0.20$; $Chi^2 = 61.97$, df = 37 (P = 0.006); $I^2 = 40\%$						
Test for overall effect: Z = 7.33 (P < 0.00001)						
Test for subgroup differences: $Chi^2 = 2.72$, df = 1 (P = 0.10), $I^2 = 63.2\%$						

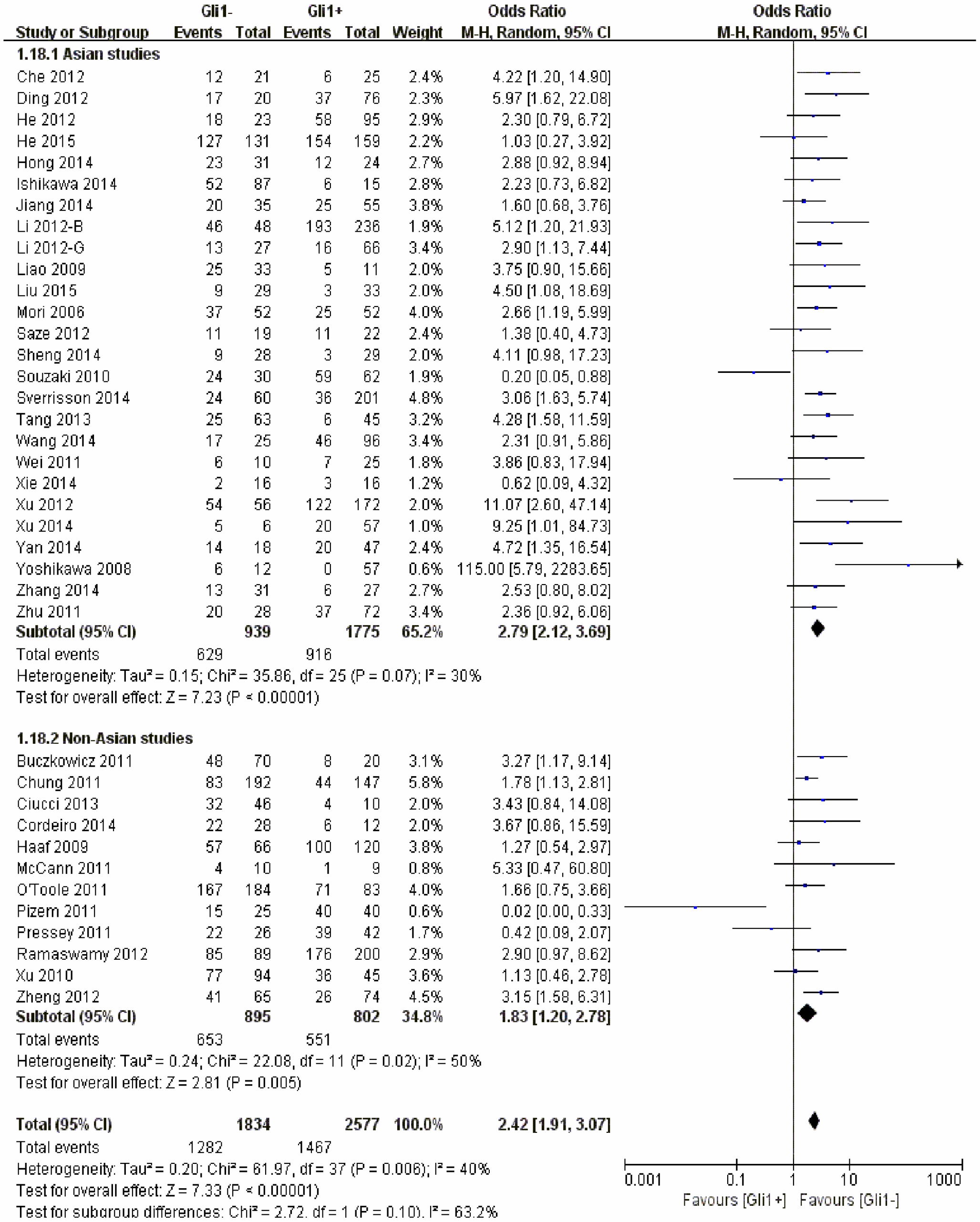

Figure S8

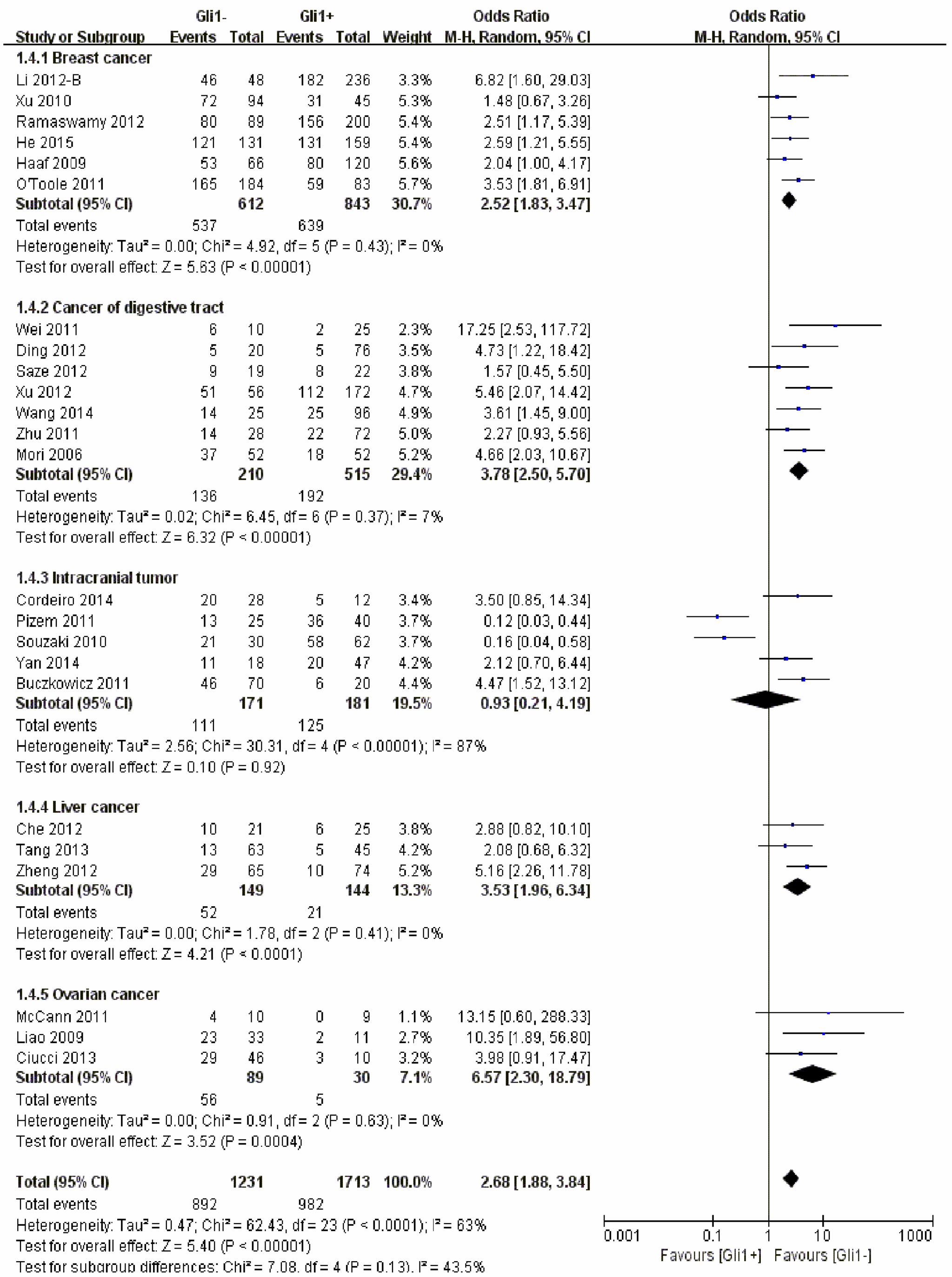

Study or Subgroup	Gli1- Events	Gli1- Total	Gli1+ Events	Gli1+ Total	Weight	Odds Ratio M-H, Random, 95% CI
**1.4.1 Breast cancer**						
Li 2012-B	46	48	182	236	3.3%	6.82 [1.60, 29.03]
Xu 2010	72	94	31	45	5.3%	1.48 [0.67, 3.26]
Ramaswamy 2012	80	89	156	200	5.4%	2.51 [1.17, 5.39]
He 2015	121	131	131	159	5.4%	2.59 [1.21, 5.55]
Haaf 2009	53	66	80	120	5.6%	2.04 [1.00, 4.17]
O'Toole 2011	165	184	59	83	5.7%	3.53 [1.81, 6.91]
**Subtotal (95% CI)**		**612**		**843**	**30.7%**	**2.52 [1.83, 3.47]**
Total events	537		639			
Heterogeneity: Tau² = 0.00; Chi² = 4.92, df = 5 (P = 0.43); I² = 0%						
Test for overall effect: Z = 5.63 (P < 0.00001)						
**1.4.2 Cancer of digestive tract**						
Wei 2011	6	10	2	25	2.3%	17.25 [2.53, 117.72]
Ding 2012	5	20	5	76	3.5%	4.73 [1.22, 18.42]
Saze 2012	9	19	8	22	3.8%	1.57 [0.45, 5.50]
Xu 2012	51	56	112	172	4.7%	5.46 [2.07, 14.42]
Wang 2014	14	25	25	96	4.9%	3.61 [1.45, 9.00]
Zhu 2011	14	28	22	72	5.0%	2.27 [0.93, 5.56]
Mori 2006	37	52	18	52	5.2%	4.66 [2.03, 10.67]
**Subtotal (95% CI)**		**210**		**515**	**29.4%**	**3.78 [2.50, 5.70]**
Total events	136		192			
Heterogeneity: Tau² = 0.02; Chi² = 6.45, df = 6 (P = 0.37); I² = 7%						
Test for overall effect: Z = 6.32 (P < 0.00001)						
**1.4.3 Intracranial tumor**						
Cordeiro 2014	20	28	5	12	3.4%	3.50 [0.85, 14.34]
Pizem 2011	13	25	36	40	3.7%	0.12 [0.03, 0.44]
Souzaki 2010	21	30	58	62	3.7%	0.16 [0.04, 0.58]
Yan 2014	11	18	20	47	4.2%	2.12 [0.70, 6.44]
Buczkowicz 2011	46	70	6	20	4.4%	4.47 [1.52, 13.12]
**Subtotal (95% CI)**		**171**		**181**	**19.5%**	**0.93 [0.21, 4.19]**
Total events	111		125			
Heterogeneity: Tau² = 2.56; Chi² = 30.31, df = 4 (P < 0.00001); I² = 87%						
Test for overall effect: Z = 0.10 (P = 0.92)						
**1.4.4 Liver cancer**						
Che 2012	10	21	6	25	3.8%	2.88 [0.82, 10.10]
Tang 2013	13	63	5	45	4.2%	2.08 [0.68, 6.32]
Zheng 2012	29	65	10	74	5.2%	5.16 [2.26, 11.78]
**Subtotal (95% CI)**		**149**		**144**	**13.3%**	**3.53 [1.96, 6.34]**
Total events	52		21			
Heterogeneity: Tau² = 0.00; Chi² = 1.78, df = 2 (P = 0.41); I² = 0%						
Test for overall effect: Z = 4.21 (P < 0.0001)						
**1.4.5 Ovarian cancer**						
McCann 2011	4	10	0	9	1.1%	13.15 [0.60, 288.33]
Liao 2009	23	33	2	11	2.7%	10.35 [1.89, 56.80]
Ciucci 2013	29	46	3	10	3.2%	3.98 [0.91, 17.47]
**Subtotal (95% CI)**		**89**		**30**	**7.1%**	**6.57 [2.30, 18.79]**
Total events	56		5			
Heterogeneity: Tau² = 0.00; Chi² = 0.91, df = 2 (P = 0.63); I² = 0%						
Test for overall effect: Z = 3.52 (P = 0.0004)						
**Total (95% CI)**		**1231**		**1713**	**100.0%**	**2.68 [1.88, 3.84]**
Total events	892		982			
Heterogeneity: Tau² = 0.47; Chi² = 62.43, df = 23 (P < 0.0001); I² = 63%						
Test for overall effect: Z = 5.40 (P < 0.00001)						
Test for subgroup differences: Chi² = 7.08, df = 4 (P = 0.13), I² = 43.5%						

Figure S9

Study or Subgroup	Gli1- Events	Gli1- Total	Gli1+ Events	Gli1+ Total	Weight	Odds Ratio M-H, Random, 95% CI
**1.9.2 Advanced stages (TNM II-IV)**						
Ciucci 2013	29	46	3	10	3.9%	3.98 [0.91, 17.47]
Ishikawa 2014	36	87	3	15	4.3%	2.82 [0.74, 10.73]
McCann 2011	4	10	0	9	1.3%	13.15 [0.60, 288.33]
Yan 2014	11	18	20	47	5.2%	2.12 [0.70, 6.44]
**Subtotal (95% CI)**		**161**		**81**	**14.7%**	**2.95 [1.44, 6.06]**
Total events	80		26			
Heterogeneity: Tau² = 0.00; Chi² = 1.41, df = 3 (P = 0.70); I² = 0%						
Test for overall effect: Z = 2.95 (P = 0.003)						
**1.9.3 Pre-terminal stages (TNM I-III)**						
Li 2012-B	46	48	182	236	4.0%	6.82 [1.60, 29.03]
Xu 2012	51	56	112	172	5.7%	5.46 [2.07, 14.42]
**Subtotal (95% CI)**		**104**		**408**	**9.7%**	**5.85 [2.61, 13.11]**
Total events	97		294			
Heterogeneity: Tau² = 0.00; Chi² = 0.06, df = 1 (P = 0.80); I² = 0%						
Test for overall effect: Z = 4.30 (P < 0.0001)						
**1.9.4 All stages (TNM I-IV)**						
Ding 2012	5	20	5	76	4.3%	4.73 [1.22, 18.42]
Hong 2014	20	31	5	24	4.7%	6.91 [2.02, 23.63]
Jiang 2014	20	35	24	55	6.2%	1.72 [0.73, 4.05]
Li 2012-G	4	27	5	66	4.1%	2.12 [0.52, 8.60]
Liao 2009	23	33	2	11	3.3%	10.35 [1.89, 56.80]
Mori 2006	37	52	18	52	6.4%	4.66 [2.03, 10.67]
Pressey 2011	21	26	39	42	3.8%	0.32 [0.07, 1.49]
Saze 2012	9	19	8	22	4.6%	1.57 [0.45, 5.50]
Souzaki 2010	21	30	58	62	4.5%	0.16 [0.04, 0.58]
Sverrisson 2014	20	60	25	201	7.0%	3.52 [1.78, 6.95]
Tang 2013	13	63	5	45	5.2%	2.08 [0.68, 6.32]
Wang 2014	14	25	25	96	6.0%	3.61 [1.45, 9.00]
Wei 2011	6	10	2	25	2.8%	17.25 [2.53, 117.72]
Xu 2010	72	94	31	45	6.5%	1.48 [0.67, 3.26]
Zhu 2011	14	28	22	72	6.1%	2.27 [0.93, 5.56]
**Subtotal (95% CI)**		**553**		**894**	**75.5%**	**2.37 [1.46, 3.85]**
Total events	299		274			
Heterogeneity: Tau² = 0.57; Chi² = 41.55, df = 14 (P = 0.0001); I² = 66%						
Test for overall effect: Z = 3.49 (P = 0.0005)						
**Total (95% CI)**		**818**		**1383**	**100.0%**	**2.71 [1.84, 4.00]**
Total events	476		594			
Heterogeneity: Tau² = 0.44; Chi² = 47.15, df = 20 (P = 0.0006); I² = 58%						
Test for overall effect: Z = 5.04 (P < 0.00001)						
Test for subgroup differences: Chi² = 3.55, df = 2 (P = 0.17), I² = 43.6%						

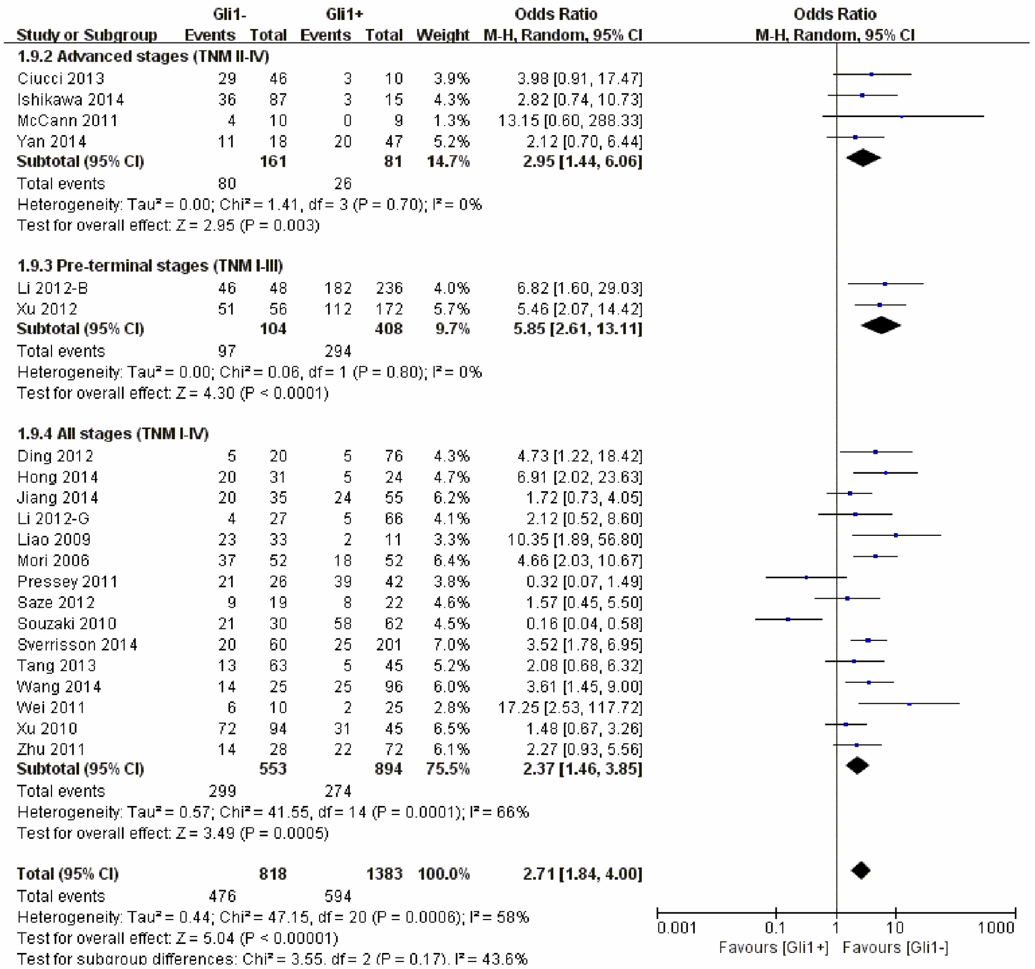

Figure S10

Study or Subgroup	Gli1- Events	Gli1- Total	Gli1+ Events	Gli1+ Total	Weight	Odds Ratio M-H, Random, 95% CI
**1.11.3 Mean age > 60**						
Hong 2014	20	31	5	24	5.9%	6.91 [2.02, 23.63]
Ishikawa 2014	36	87	3	15	5.4%	2.82 [0.74, 10.73]
Jiang 2014	20	35	24	55	7.9%	1.72 [0.73, 4.05]
McCann 2011	4	10	0	9	1.6%	13.15 [0.60, 288.33]
Mori 2006	37	52	18	52	8.1%	4.66 [2.03, 10.67]
Saze 2012	9	19	8	22	5.8%	1.57 [0.45, 5.50]
Wang 2014	14	25	25	96	7.6%	3.61 [1.45, 9.00]
Wei 2011	6	10	2	25	3.5%	17.25 [2.53, 117.72]
**Subtotal (95% CI)**		**269**		**298**	**45.9%**	**3.54 [2.19, 5.72]**
Total events	146		85			
Heterogeneity: Tau² = 0.11; Chi² = 9.29, df = 7 (P = 0.23); I² = 25%						
Test for overall effect: Z = 5.15 (P < 0.00001)						
**1.11.4 Mean age < 60**						
Buczkowicz 2011	46	70	6	20	6.7%	4.47 [1.52, 13.12]
Che 2012	10	21	6	25	5.8%	2.88 [0.82, 10.10]
Ciucci 2013	29	46	3	10	4.8%	3.98 [0.91, 17.47]
Haaf 2009	53	66	80	120	8.8%	2.04 [1.00, 4.17]
Li 2012-B	46	48	182	236	5.0%	6.82 [1.60, 29.03]
O'Toole 2011	165	184	59	83	9.0%	3.53 [1.81, 6.91]
Pizem 2011	13	25	36	40	5.6%	0.12 [0.03, 0.44]
Ramaswamy 2012	80	89	156	200	8.5%	2.51 [1.17, 5.39]
**Subtotal (95% CI)**		**549**		**734**	**54.1%**	**2.32 [1.17, 4.58]**
Total events	442		528			
Heterogeneity: Tau² = 0.66; Chi² = 25.77, df = 7 (P = 0.0006); I² = 73%						
Test for overall effect: Z = 2.42 (P = 0.02)						
**Total (95% CI)**		**818**		**1032**	**100.0%**	**2.89 [1.89, 4.43]**
Total events	588		613			
Heterogeneity: Tau² = 0.41; Chi² = 36.63, df = 15 (P = 0.001); I² = 59%						
Test for overall effect: Z = 4.88 (P < 0.00001)						
Test for subgroup differences: Chi² = 0.99, df = 1 (P = 0.32), I² = 0%						

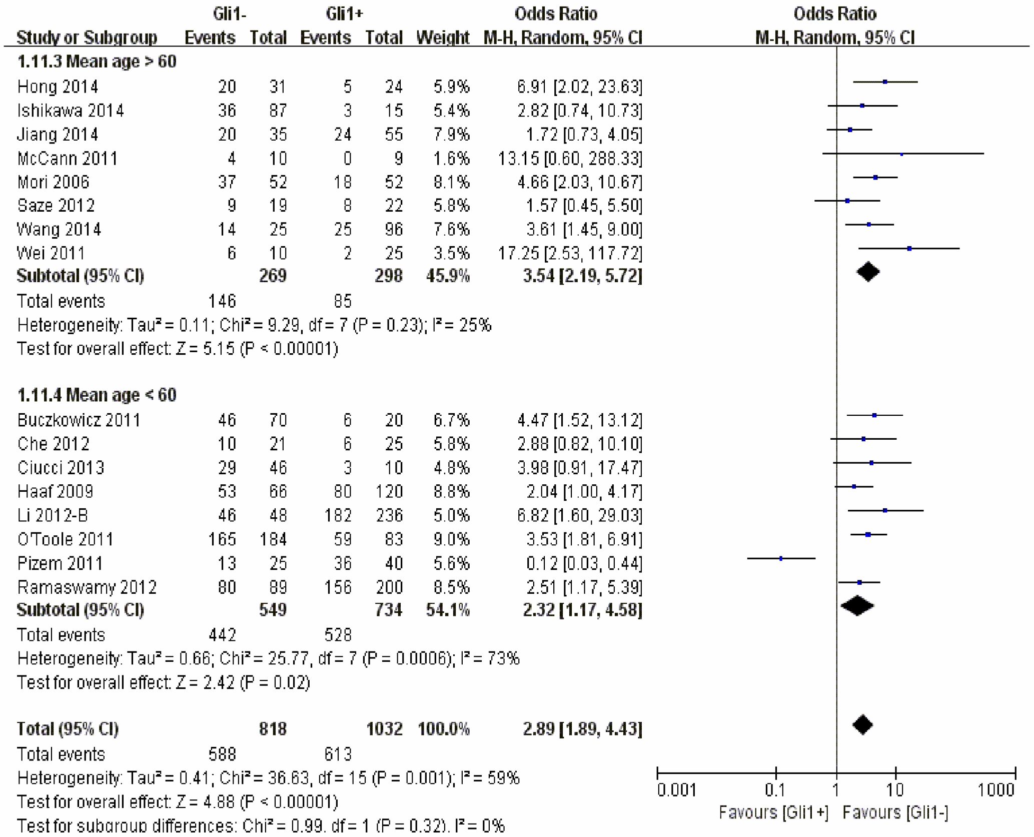

Figure S11

Study or Subgroup	Gli1- Events	Gli1- Total	Gli1+ Events	Gli1+ Total	Weight	Odds Ratio M-H, Random, 95% CI
**1.13.1 PCR**						
Che 2012	10	21	6	25	2.9%	2.88 [0.82, 10.10]
Cordeiro 2014	20	28	5	12	2.5%	3.50 [0.85, 14.34]
Hong 2014	20	31	5	24	2.9%	6.91 [2.02, 23.63]
Ishikawa 2014	36	87	3	15	2.7%	2.82 [0.74, 10.73]
McCann 2011	4	10	0	9	0.8%	13.15 [0.60, 288.33]
Pressey 2011	21	26	39	42	2.3%	0.32 [0.07, 1.49]
Saze 2012	9	19	8	22	2.9%	1.57 [0.45, 5.50]
Zheng 2012	29	65	10	74	4.1%	5.16 [2.26, 11.78]
**Subtotal (95% CI)**		**287**		**223**	**21.1%**	**2.85 [1.48, 5.49]**
Total events	149		76			
Heterogeneity: Tau² = 0.41; Chi² = 13.55, df = 7 (P = 0.06); I² = 48%						
Test for overall effect: Z = 3.13 (P = 0.002)						
**1.13.2 Immunohistochemistry (IHC)**						
Buczkowicz 2011	46	70	6	20	3.3%	4.47 [1.52, 13.12]
Chung 2011	65	192	26	147	5.1%	2.38 [1.42, 4.00]
Ciucci 2013	29	46	3	10	2.4%	3.98 [0.91, 17.47]
Ding 2012	5	20	5	76	2.6%	4.73 [1.22, 18.42]
Haaf 2009	53	66	80	120	4.4%	2.04 [1.00, 4.17]
He 2015	121	131	131	159	4.3%	2.59 [1.21, 5.55]
Jiang 2014	20	35	5	24	3.0%	5.07 [1.54, 16.67]
Li 2012-B	46	48	182	236	2.5%	6.82 [1.60, 29.03]
Li 2012-G	4	27	5	66	2.6%	2.12 [0.52, 8.60]
Liao 2009	23	33	2	11	2.0%	10.35 [1.89, 56.80]
Mori 2006	37	52	18	52	4.1%	4.66 [2.03, 10.67]
O'Toole 2011	165	184	59	83	4.6%	3.53 [1.81, 6.91]
Pizem 2011	13	25	36	40	2.8%	0.12 [0.03, 0.44]
Ramaswamy 2012	80	89	156	200	4.3%	2.51 [1.17, 5.39]
Souzaki 2010	21	30	58	62	2.8%	0.16 [0.04, 0.58]
Sverrisson 2014	20	60	25	201	4.5%	3.52 [1.78, 6.95]
Tang 2013	13	63	5	45	3.2%	2.08 [0.68, 6.32]
Wang 2014	14	25	25	96	3.8%	3.61 [1.45, 9.00]
Wei 2011	6	10	2	25	1.7%	17.25 [2.53, 117.72]
Xu 2010	72	94	31	45	4.2%	1.48 [0.67, 3.26]
Xu 2012	51	56	112	172	3.6%	5.46 [2.07, 14.42]
Yan 2014	11	18	20	47	3.2%	2.12 [0.70, 6.44]
Zhu 2011	14	28	22	72	3.9%	2.27 [0.93, 5.56]
**Subtotal (95% CI)**		**1402**		**2009**	**78.9%**	**2.63 [1.88, 3.69]**
Total events	929		1014			
Heterogeneity: Tau² = 0.39; Chi² = 60.17, df = 22 (P < 0.0001); I² = 63%						
Test for overall effect: Z = 5.65 (P < 0.00001)						
**Total (95% CI)**		**1689**		**2232**	**100.0%**	**2.68 [1.99, 3.60]**
Total events	1078		1090			
Heterogeneity: Tau² = 0.38; Chi² = 74.12, df = 30 (P < 0.0001); I² = 60%						
Test for overall effect: Z = 6.55 (P < 0.00001)						
Test for subgroup differences: Chi² = 0.04, df = 1 (P = 0.84), I² = 0%						

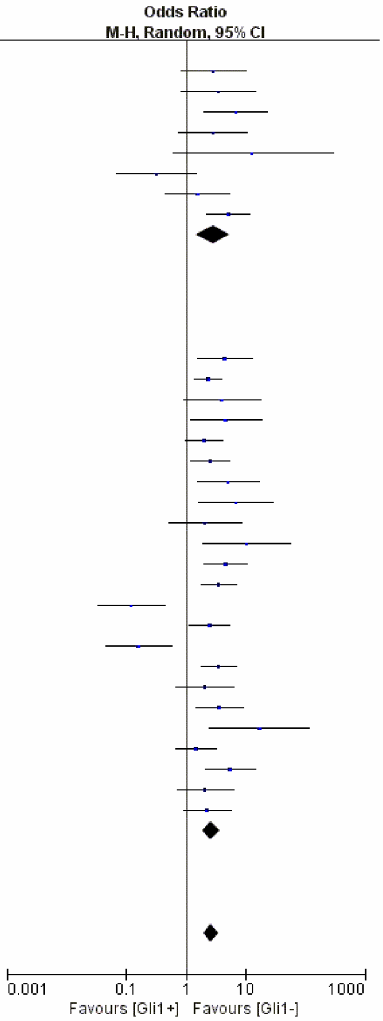

Figure S12

Study or Subgroup	Gli1- Events	Gli1- Total	Gli1+ Events	Gli1+ Total	Weight	Odds Ratio M-H, Random, 95% CI
**1.15.1 Cytoplasmic expression**						
Jiang 2014	20	35	24	55	5.0%	1.72 [0.73, 4.05]
Liao 2009	23	33	2	11	2.5%	10.35 [1.89, 56.80]
Souzaki 2010	21	30	58	62	3.5%	0.16 [0.04, 0.58]
**Subtotal (95% CI)**		**98**		**128**	**11.0%**	**1.34 [0.17, 10.48]**
Total events	64		84			
Heterogeneity: Tau² = 2.85; Chi² = 16.32, df = 2 (P = 0.0003); I² = 88%						
Test for overall effect: Z = 0.28 (P = 0.78)						
**1.15.2 Nuclear expression**						
Buczkowicz 2011	46	70	6	20	4.2%	4.47 [1.52, 13.12]
Ciucci 2013	29	46	3	10	3.0%	3.98 [0.91, 17.47]
Ding 2012	5	20	5	76	3.3%	4.73 [1.22, 18.42]
Haaf 2009	53	66	80	120	5.5%	2.04 [1.00, 4.17]
Li 2012-B	46	48	182	236	3.1%	6.82 [1.60, 29.03]
Li 2012-G	4	27	5	66	3.2%	2.12 [0.52, 8.60]
Mori 2006	37	52	18	52	5.1%	4.66 [2.03, 10.67]
Pizem 2011	13	25	36	40	3.5%	0.12 [0.03, 0.44]
Wei 2011	6	10	2	25	2.1%	17.25 [2.53, 117.72]
Xu 2012	51	56	112	172	4.6%	5.46 [2.07, 14.42]
Zhu 2011	14	28	22	72	4.8%	2.27 [0.93, 5.56]
**Subtotal (95% CI)**		**448**		**889**	**42.4%**	**3.00 [1.61, 5.58]**
Total events	304		471			
Heterogeneity: Tau² = 0.73; Chi² = 33.39, df = 10 (P = 0.0002); I² = 70%						
Test for overall effect: Z = 3.45 (P = 0.0006)						
**1.15.3 Unspecific expression**						
Chung 2011	65	192	26	147	6.3%	2.38 [1.42, 4.00]
He 2015	121	131	131	159	5.4%	2.59 [1.21, 5.55]
O'Toole 2011	165	184	59	83	5.7%	3.53 [1.81, 6.91]
Ramaswamy 2012	80	89	156	200	5.3%	2.51 [1.17, 5.39]
Sverrisson 2014	20	60	25	201	5.7%	3.52 [1.78, 6.95]
Tang 2013	13	63	5	45	4.1%	2.08 [0.68, 6.32]
Wang 2014	14	25	25	96	4.8%	3.61 [1.45, 9.00]
Xu 2010	72	94	31	45	5.2%	1.48 [0.67, 3.26]
Yan 2014	11	18	20	47	4.1%	2.12 [0.70, 6.44]
**Subtotal (95% CI)**		**856**		**1023**	**46.6%**	**2.61 [2.03, 3.36]**
Total events	561		478			
Heterogeneity: Tau² = 0.00; Chi² = 4.43, df = 8 (P = 0.82); I² = 0%						
Test for overall effect: Z = 7.52 (P < 0.00001)						
**Total (95% CI)**		**1402**		**2040**	**100.0%**	**2.52 [1.81, 3.50]**
Total events	929		1033			
Heterogeneity: Tau² = 0.38; Chi² = 59.79, df = 22 (P < 0.0001); I² = 63%						
Test for overall effect: Z = 5.47 (P < 0.00001)						
Test for subgroup differences: Chi² = 0.58, df = 2 (P = 0.75), I² = 0%						

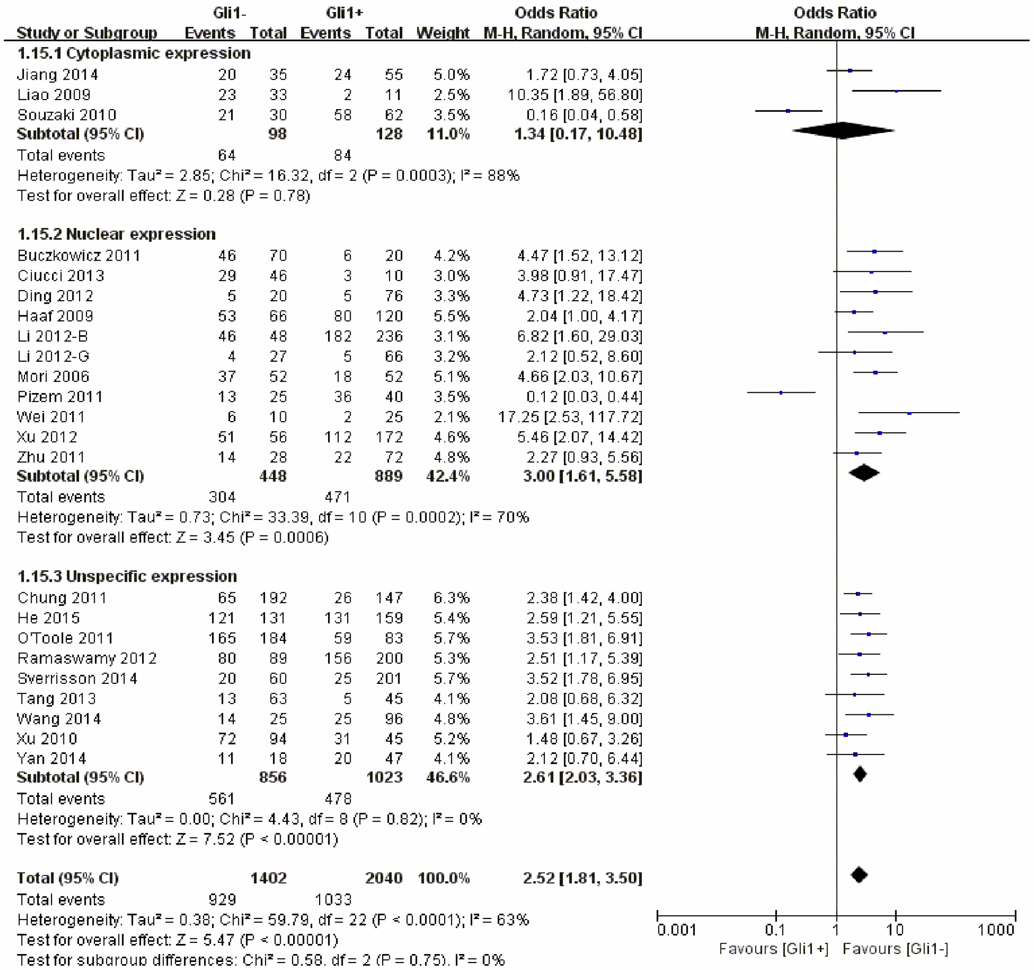

Figure S13

Study or Subgroup	Gli1- Events	Gli1- Total	Gli1+ Events	Gli1+ Total	Weight	Odds Ratio M-H, Random, 95% CI
**1.17.1 Sample-size > 90**						
Buczkowicz 2011	46	70	6	20	3.3%	4.47 [1.52, 13.12]
Chung 2011	65	192	26	147	5.0%	2.38 [1.42, 4.00]
Ding 2012	5	20	5	76	2.6%	4.73 [1.22, 18.42]
Haaf 2009	53	66	80	120	4.4%	2.04 [1.00, 4.17]
He 2015	121	131	131	159	4.3%	2.59 [1.21, 5.55]
Ishikawa 2014	36	87	3	15	2.7%	2.82 [0.74, 10.73]
Jiang 2014	20	35	24	55	4.0%	1.72 [0.73, 4.05]
Li 2012-B	46	48	182	236	2.4%	6.82 [1.60, 29.03]
Li 2012-G	4	27	5	66	2.5%	2.12 [0.52, 8.60]
Mori 2006	37	52	18	52	4.0%	4.66 [2.03, 10.67]
O'Toole 2011	165	184	59	83	4.6%	3.53 [1.81, 6.91]
Ramaswamy 2012	80	89	156	200	4.2%	2.51 [1.17, 5.39]
Souzaki 2010	21	30	58	62	2.8%	0.16 [0.04, 0.58]
Sverrisson 2014	20	60	25	201	4.5%	3.52 [1.78, 6.95]
Tang 2013	13	63	5	45	3.2%	2.08 [0.68, 6.32]
Wang 2014	14	25	25	96	3.8%	3.61 [1.45, 9.00]
Xu 2010	72	94	31	45	4.2%	1.48 [0.67, 3.26]
Xu 2012	51	56	112	172	3.6%	5.46 [2.07, 14.42]
Zheng 2012	29	65	10	74	4.0%	5.16 [2.26, 11.78]
Zhu 2011	14	28	22	72	3.8%	2.27 [0.93, 5.56]
**Subtotal (95% CI)**		**1422**		**1996**	**73.9%**	**2.71 [2.07, 3.53]**
Total events	912		983			
Heterogeneity: Tau² = 0.15; Chi² = 34.00, df = 19 (P = 0.02); I² = 44%						
Test for overall effect: Z = 7.32 (P < 0.00001)						
**1.17.2 Sample-size < 90**						
Che 2012	10	21	6	25	2.8%	2.88 [0.82, 10.10]
Ciucci 2013	29	46	3	10	2.4%	3.98 [0.91, 17.47]
Cordeiro 2014	20	28	5	12	2.5%	3.50 [0.85, 14.34]
Hong 2014	20	31	5	24	2.9%	6.91 [2.02, 23.63]
Liao 2009	23	33	2	11	2.0%	10.35 [1.89, 56.80]
McCann 2011	4	10	0	9	0.8%	13.15 [0.60, 288.33]
Pizem 2011	13	25	36	40	2.7%	0.12 [0.03, 0.44]
Pressey 2011	21	26	39	42	2.3%	0.32 [0.07, 1.49]
Saze 2012	9	19	8	22	2.8%	1.57 [0.45, 5.50]
Wei 2011	6	10	2	25	1.7%	17.25 [2.53, 117.72]
Yan 2014	11	18	20	47	3.2%	2.12 [0.70, 6.44]
**Subtotal (95% CI)**		**267**		**267**	**26.1%**	**2.49 [1.04, 5.95]**
Total events	166		126			
Heterogeneity: Tau² = 1.55; Chi² = 39.12, df = 10 (P < 0.0001); I² = 74%						
Test for overall effect: Z = 2.05 (P = 0.04)						
**Total (95% CI)**		**1689**		**2263**	**100.0%**	**2.58 [1.93, 3.46]**
Total events	1078		1109			
Heterogeneity: Tau² = 0.37; Chi² = 73.97, df = 30 (P < 0.0001); I² = 59%						
Test for overall effect: Z = 6.38 (P < 0.00001)						
Test for subgroup differences: Chi² = 0.03, df = 1 (P = 0.86), I² = 0%						

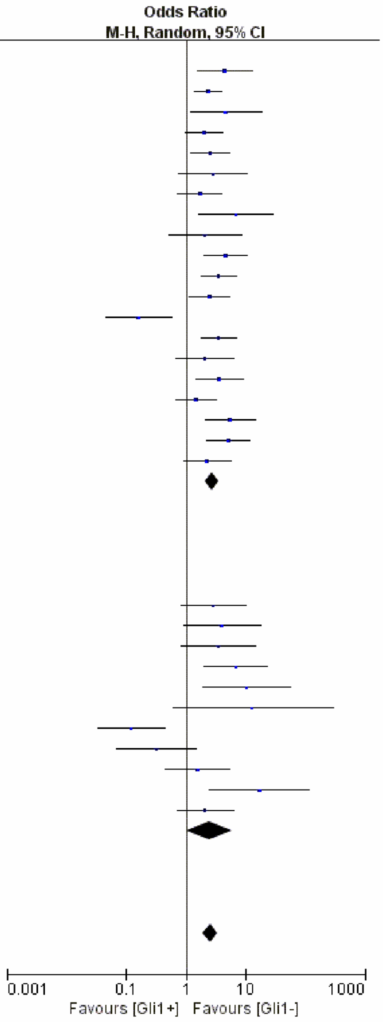

Figure S14

Study or Subgroup	Gli1- Events	Gli1- Total	Gli1+ Events	Gli1+ Total	Weight	Odds Ratio M-H, Random, 95% CI
**1.19.1 Asian studies**						
Che 2012	10	21	6	25	2.8%	2.88 [0.82, 10.10]
Ding 2012	5	20	5	76	2.6%	4.73 [1.22, 18.42]
He 2015	121	131	131	159	4.3%	2.59 [1.21, 5.55]
Hong 2014	20	31	5	24	2.9%	6.91 [2.02, 23.63]
Ishikawa 2014	36	87	3	15	2.7%	2.82 [0.74, 10.73]
Jiang 2014	20	35	24	55	4.0%	1.72 [0.73, 4.05]
Li 2012-B	46	48	182	236	2.4%	6.82 [1.60, 29.03]
Li 2012-G	4	27	5	66	2.5%	2.12 [0.52, 8.60]
Liao 2009	23	33	2	11	2.0%	10.35 [1.89, 56.80]
Mori 2006	37	52	18	52	4.0%	4.66 [2.03, 10.67]
Saze 2012	9	19	8	22	2.8%	1.57 [0.45, 5.50]
Souzaki 2010	21	30	58	62	2.8%	0.16 [0.04, 0.58]
Sverrisson 2014	20	60	25	201	4.5%	3.52 [1.78, 6.95]
Tang 2013	13	63	5	45	3.2%	2.08 [0.68, 6.32]
Wang 2014	14	25	25	96	3.8%	3.61 [1.45, 9.00]
Wei 2011	6	10	2	25	1.7%	17.25 [2.53, 117.72]
Xu 2012	51	56	112	172	3.6%	5.46 [2.07, 14.42]
Yan 2014	11	18	20	47	3.2%	2.12 [0.70, 6.44]
Zhu 2011	14	28	22	72	3.8%	2.27 [0.93, 5.56]
**Subtotal (95% CI)**		**794**		**1461**	**59.6%**	**2.94 [2.06, 4.20]**
Total events	481		658			
Heterogeneity: Tau² = 0.29; Chi² = 35.82, df = 18 (P = 0.007); I² = 50%						
Test for overall effect: Z = 5.96 (P < 0.00001)						
**1.19.2 Non-Asian studies**						
Buczkowicz 2011	46	70	6	20	3.3%	4.47 [1.52, 13.12]
Chung 2011	65	192	26	147	5.0%	2.38 [1.42, 4.00]
Ciucci 2013	29	46	3	10	2.4%	3.98 [0.91, 17.47]
Cordeiro 2014	20	28	5	12	2.5%	3.50 [0.85, 14.34]
Haaf 2009	53	66	80	120	4.4%	2.04 [1.00, 4.17]
McCann 2011	4	10	0	9	0.8%	13.15 [0.60, 288.33]
O'Toole 2011	165	184	59	83	4.6%	3.53 [1.81, 6.91]
Pizem 2011	13	25	36	40	2.7%	0.12 [0.03, 0.44]
Pressey 2011	21	26	39	42	2.3%	0.32 [0.07, 1.49]
Ramaswamy 2012	80	89	156	200	4.2%	2.51 [1.17, 5.39]
Xu 2010	72	94	31	45	4.2%	1.48 [0.67, 3.26]
Zheng 2012	29	65	10	74	4.0%	5.16 [2.26, 11.78]
**Subtotal (95% CI)**		**895**		**802**	**40.4%**	**2.10 [1.27, 3.48]**
Total events	597		451			
Heterogeneity: Tau² = 0.49; Chi² = 36.48, df = 11 (P = 0.0001); I² = 70%						
Test for overall effect: Z = 2.89 (P = 0.004)						
**Total (95% CI)**		**1689**		**2263**	**100.0%**	**2.58 [1.93, 3.46]**
Total events	1078		1109			
Heterogeneity: Tau² = 0.37; Chi² = 73.97, df = 30 (P < 0.0001); I² = 59%						
Test for overall effect: Z = 6.38 (P < 0.00001)						
Test for subgroup differences: Chi² = 1.15, df = 1 (P = 0.28), I² = 12.7%						

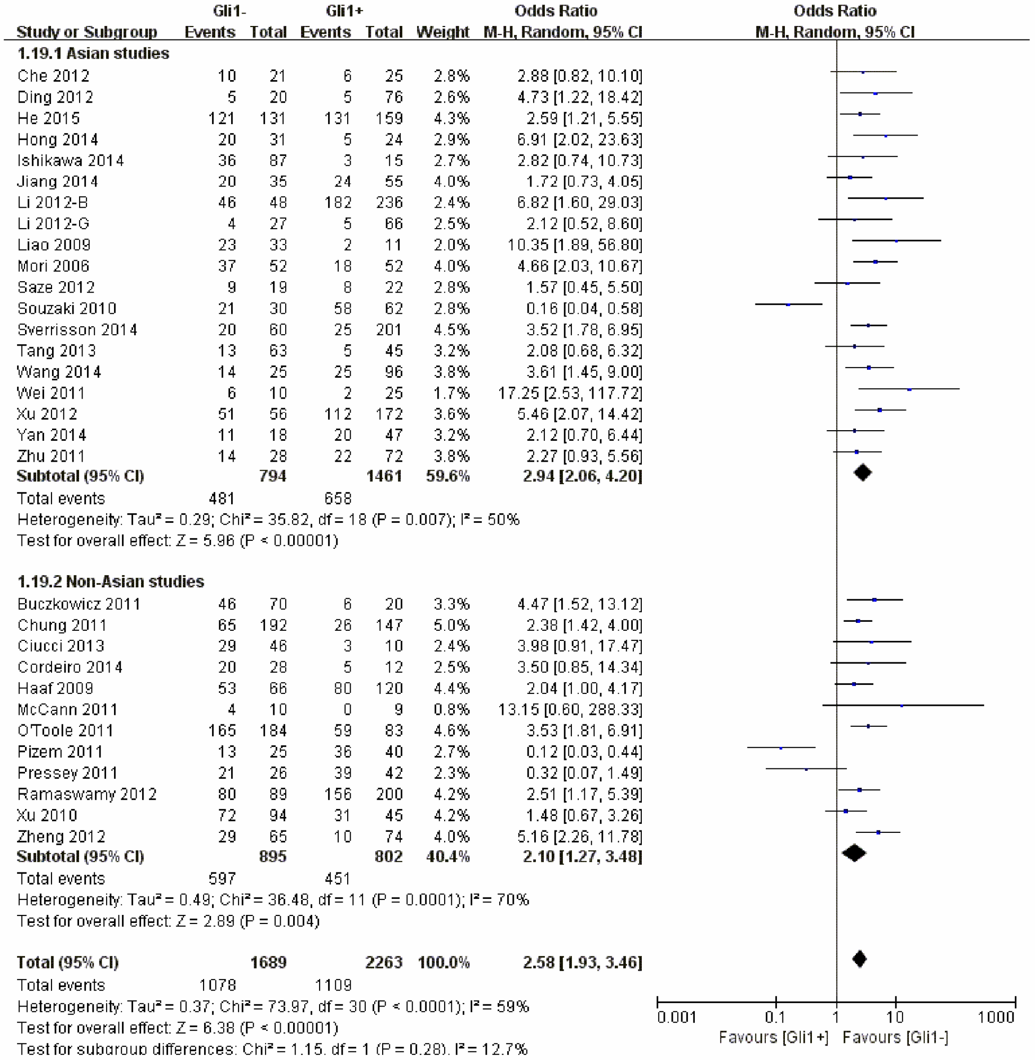

Figure S15

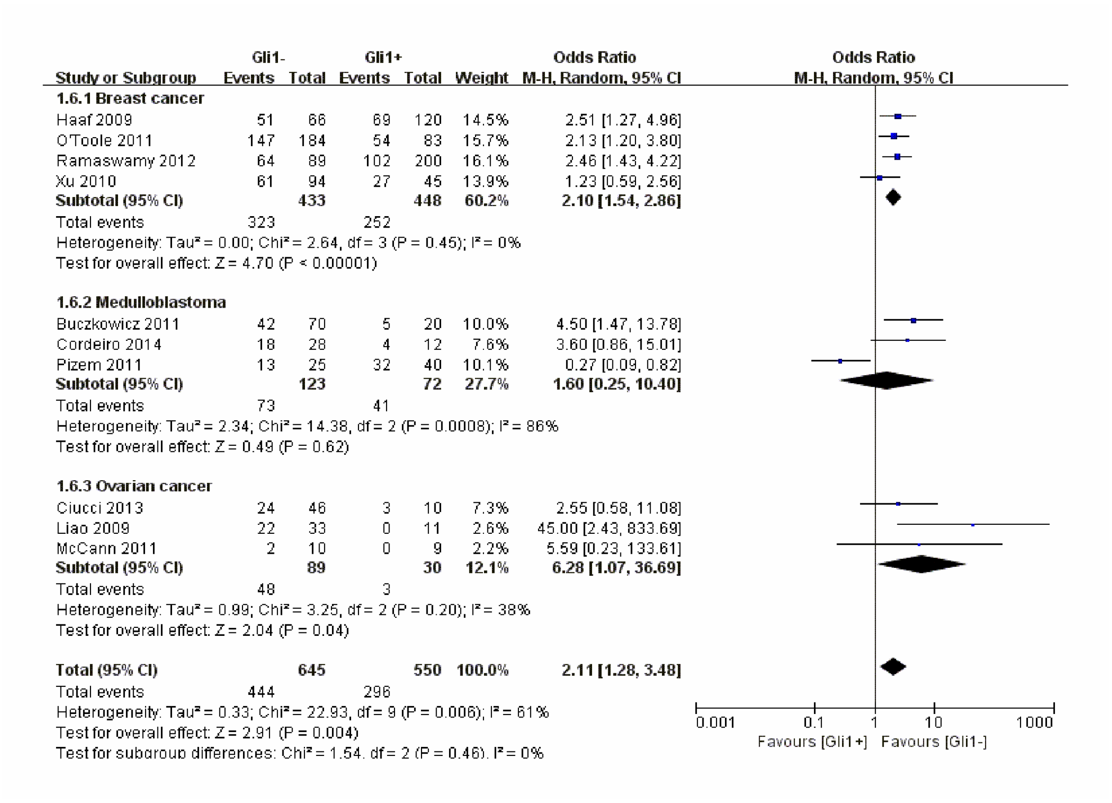

Study or Subgroup	Gli1- Events	Gli1- Total	Gli1+ Events	Gli1+ Total	Weight	Odds Ratio M-H, Random, 95% CI
**1.6.1 Breast cancer**						
Haaf 2009	51	66	69	120	14.5%	2.51 [1.27, 4.96]
O'Toole 2011	147	184	54	83	15.7%	2.13 [1.20, 3.80]
Ramaswamy 2012	64	89	102	200	16.1%	2.46 [1.43, 4.22]
Xu 2010	61	94	27	45	13.9%	1.23 [0.59, 2.56]
**Subtotal (95% CI)**		**433**		**448**	**60.2%**	**2.10 [1.54, 2.86]**
Total events	323		252			
Heterogeneity: Tau² = 0.00; Chi² = 2.64, df = 3 (P = 0.45); I² = 0%						
Test for overall effect: Z = 4.70 (P < 0.00001)						
**1.6.2 Medulloblastoma**						
Buczkowicz 2011	42	70	5	20	10.0%	4.50 [1.47, 13.78]
Cordeiro 2014	18	28	4	12	7.6%	3.60 [0.86, 15.01]
Pizem 2011	13	25	32	40	10.1%	0.27 [0.09, 0.82]
**Subtotal (95% CI)**		**123**		**72**	**27.7%**	**1.60 [0.25, 10.40]**
Total events	73		41			
Heterogeneity: Tau² = 2.34; Chi² = 14.38, df = 2 (P = 0.0008); I² = 86%						
Test for overall effect: Z = 0.49 (P = 0.62)						
**1.6.3 Ovarian cancer**						
Ciucci 2013	24	46	3	10	7.3%	2.55 [0.58, 11.08]
Liao 2009	22	33	0	11	2.6%	45.00 [2.43, 833.69]
McCann 2011	2	10	0	9	2.2%	5.59 [0.23, 133.61]
**Subtotal (95% CI)**		**89**		**30**	**12.1%**	**6.28 [1.07, 36.69]**
Total events	48		3			
Heterogeneity: Tau² = 0.99; Chi² = 3.25, df = 2 (P = 0.20); I² = 38%						
Test for overall effect: Z = 2.04 (P = 0.04)						
**Total (95% CI)**		**645**		**550**	**100.0%**	**2.11 [1.28, 3.48]**
Total events	444		296			
Heterogeneity: Tau² = 0.33; Chi² = 22.93, df = 9 (P = 0.006); I² = 61%						
Test for overall effect: Z = 2.91 (P = 0.004)						
Test for subgroup differences: Chi² = 1.54, df = 2 (P = 0.46), I² = 0%						

Supplementary Figure S1- The forest plot of 3-year overall survival in terms of different cancer types

Supplementary Figure S2- The forest plot of 3-year overall survival in terms of different TNM stages

Supplementary Figure S3- The forest plot of 3-year overall survival in terms of different mean-age

Supplementary Figure S4- The forest plot of 3-year overall survival in terms of different detection approaches

Supplementary Figure S5- The forest plot of 3-year overall survival in terms of different subcellular localizations

Supplementary Figure S6- The forest plot of 3-year overall survival in terms of different sample-size

Supplementary Figure S7- The forest plot of 3-year overall survival in terms of different source regions

Supplementary Figure S8- The forest plot of 5-year overall survival in terms of different cancer types

Supplementary Figure S9- The forest plot of 5-year overall survival in terms of different TNM stages

Supplementary Figure S10- The forest plot of 5-year overall survival in terms of different mean-age

Supplementary Figure S11- The forest plot of 5-year overall survival in terms of different detection approaches

Supplementary Figure S12- The forest plot of 5-year overall survival in terms of different subcellular localizations

Supplementary Figure S13- The forest plot of 5-year overall survival in terms of different sample-size

Supplementary Figure S14- The forest plot of 5-year overall survival in terms of different source regions

Supplementary Figure S15- The forest plot of 10-year overall survival in terms of different cancer types
